# PICALO: principal interaction component analysis for the identification of discrete technical, cell-type, and environmental factors that mediate eQTLs

**DOI:** 10.1186/s13059-023-03151-0

**Published:** 2024-01-22

**Authors:** Martijn Vochteloo, Patrick Deelen, Britt Vink, BIOS Consortium, Ellen A. Tsai, Heiko Runz, Sergio Andreu-Sánchez, Jingyuan Fu, Alexandra Zhernakova, Harm-Jan Westra, Lude Franke

**Affiliations:** 1 Department of Genetics, University Medical Center Groningen, University of Groningen, Groningen, The Netherlands; 2 Oncode Institute, Utrecht, The Netherlands; 3 Institute for Life Science & Technology, Hanze University of Applied Sciences, Groningen, The Netherlands; 4 Translational Sciences, Research and Development, Biogen, Cambridge, MA USA; 5 Department of Pediatrics, University Medical Center Groningen, University of Groningen, Groningen, The Netherlands

**Keywords:** eQTLs, Hidden variable inference, Context, Cell type, Interaction eQTLs

## Abstract

Expression quantitative trait loci (eQTL) offer insights into the regulatory mechanisms of trait-associated variants, but their effects often rely on contexts that are unknown or unmeasured. We introduce PICALO, a method for hidden variable inference of eQTL contexts. PICALO identifies and disentangles technical from biological context in heterogeneous blood and brain bulk eQTL datasets. These contexts are biologically informative and reproducible, outperforming cell counts or expression-based principal components. Furthermore, we show that RNA quality and cell type proportions interact with thousands of eQTLs. Knowledge of hidden eQTL contexts may aid in the inference of functional mechanisms underlying disease variants.

**Supplementary Information:**

The online version contains supplementary material available at 10.1186/s13059-023-03151-0.

## Background

Expression quantitative trait locus (eQTL) mapping is often used to make inferences on the transcriptional consequences of genetic variants that have been identified through genome-wide association studies (GWAS). A challenge of eQTL studies is that the regulatory potential of a variant is often context-dependent, resulting in differences in eQTL effect strengths between tissues [1], cell types [2], and stimulations [3]. This hinders proper interpretation of disease-associated variants [4]. Many different strategies have been employed to identify context-dependent eQTLs: for instance, the GTEx consortium generated data in many different tissues [5] and populations [6], and in the MetaBrain project, many brain eQTL datasets were combined to improve the ability to identify brain-dependent eQTLs [7]. Furthermore, single-cell RNA sequencing has been instrumental in identifying cell type-dependent eQTLs within the same tissue [8–10]. By using ex vivo stimulations [3, 11, 12] or case control [13, 14] comparisons of eQTL effect strength, it is also possible to identify stimulation-dependent regulatory effects. However, the number of available contexts in these studies has remained somewhat limited, leaving many context-dependent eQTLs to be discovered.

Since data generation is expensive, various computational methods have been developed that do not rely on directly measured contexts or stimulations, but instead estimate factors that influence context-dependent eQTLs. The influence of such a context on eQTL effect sizes is often determined through interaction eQTL (ieQTL) analysis [15]. This principle has been applied previously to identify cell type-dependent eQTLs, for instance, by using predicted cell count measurements in bulk data to estimate the contribution of different cell types to an eQTL effect [15, 16]. More complex models, such as sn-spMF, can detect factors representing tissue specificity of eQTL effects by using bulk data from different tissues [17]. To identify eQTLs dependent on contexts other than cell types or tissue, other genes have previously also been used in ieQTL analysis. For example, blood-based gene expression levels of other genes were used previously to identify context-dependent effects [18], some of which were related to type 1 interferon signaling. A recent study using the GTEx dataset also revealed context-dependent eQTLs that could be attributed to transcription factor levels [19]. The limitations of these methods are that not all confounding contexts might be known or easily measurable and that individual (gene expression) measurements might not be perfect proxies for specific contexts and thus can be noisy. For instance, cell type quantifications can differ, depending on the used technology and gate settings whereas measured expression levels of specific genes are unlikely to perfectly reflect environmental stimuli or transcription factor activity. Methods to infer hidden variables (e.g., principal component analysis (PCA) [20], surrogate variable analysis (SVA) [21], probabilistic estimation of expression residuals (PEER) [22], and hidden covariates with prior (HCP) [23]) can also be used to identify proxies for contexts that have not been directly measured or are not readily predicted in a bulk dataset. By applying such methods on gene expression data, it is possible to capture components that can be tested as a potential proxy of a context that might influence eQTL effect strengths. Recent systematic comparisons between these hidden variable inference methods have shown that PCA is superior to the alternative hidden variable inference methods, being easy to use, magnitudes faster, and much easier to interpret [24]. However, principal components (PCs) often capture the gene expression variance explained by a mixture of different biological and technical signals in a single component [18]. Therefore, it is often unclear how to interpret the eQTLs that interact with such PCs.

To attempt to resolve these issues, we developed Principal Interaction Component Analysis through Likelihood Optimization (PICALO), a hidden variable inference method using expectation maximization that automatically identifies and disentangles technical and biological hidden variables, referred to as principal interaction components (PIC), that serve as proxies for contexts. We applied PICALO to bulk RNA-seq eQTL datasets in the blood (*n* = 2932) and brain (*n* = 2440). We identified a set of highly informative PICs that together influence > 39% of eQTLs. The observed PICs are associated with RNA quality, cell type composition, and environmental influences and can be replicated across ancestries. We show that PICs less often capture a mixture of biological and technical contexts, as compared to expression PCs. Moreover, we show that between 62 and 64% of eQTL interactions are with technical PICs, highlighting the importance of proper correction for these effects when performing interaction analysis. As such, PICALO is a self-supervised method to identify optimal proxy variables for hidden contexts that influence eQTL effect sizes.

## Results

### PICALO identifies eQTL context PICs through optimization of the interaction log-likelihood

**Fig. 1:**
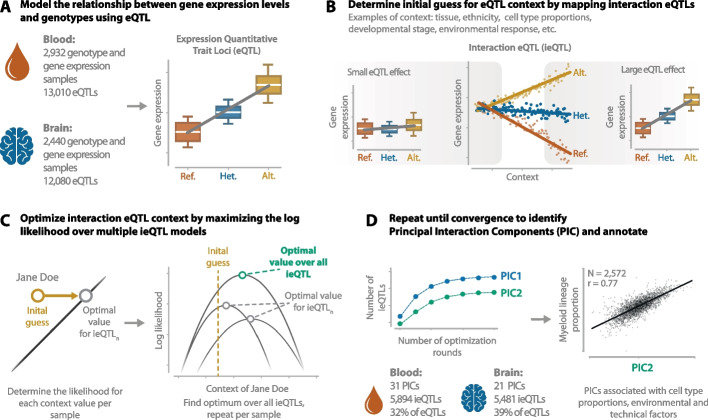
Graphical overview of the PICALO method. **A** PICALO takes eQTL data (i.e., gene expression and genotype dosage values) as input. **B** Map interactions with a starting position representing an initial guess of biological/technical context. **C** The starting position is optimized by maximizing the joint log-likelihood on a per-sample basis over multiple ieQTLs. **D** Mapping of the interactions and the subsequent optimization are repeated until convergence. The influence of the resulting principal interacting component (PIC) is regressed out from the gene expression data, and the process is repeated until no additional PICs and ieQTLs are identified. The resulting PICs capture technical and biological contexts such as cell type proportions. The illustrations shown in A, B and C are generated using dummy data

To identify the context that influences eQTL effect sizes (e.g., RNA quality, cell type composition, environmental factors), we use the genotype data and tissue expression data for known eQTLs in the relevant tissue (Fig. 1A). Using this data, PICALO identifies the biological and technical context specific to the supplied data by mapping ieQTLs (Fig. 1B) and subsequently optimizing the likelihood of the interaction terms using an expectation maximization (EM) approach (Fig. 1C). In short, a starting position for the optimization (i.e., the expectation; an initial guess of a context such as expression PCs, marker gene expression, cell proportion, etc.) is used to identify an initial set of Benjamini–Hochberg false discovery rate [25] (BH-FDR < 0.05) significant ieQTLs. If more than one possible starting position is supplied (e.g., multiple different expression PCs), the optimization is started with the starting position that had the highest number of ieQTLs. Next, the values of the starting position are adjusted to maximize the log-likelihood of the interaction model for the set of included ieQTLs (this requires at least two ieQTLs). This adjusted vector is subsequently used as an updated expectation to reidentify a set of significant ieQTLs (most often increasing the size of the set of ieQTLs that were found in the initial ieQTL identification step), after which the process repeats until the vector no longer changes and thus convergence is reached. The resulting context vector is referred to as a PIC (Fig. 1D). The gene expression data is then adjusted for this PIC and its interaction effect with eQTLs using ordinary least squares (OLS) regression, after which a second PIC can be identified. This procedure is repeated, until no additional significant ieQTLs can be found. The code for PICALO and an animated explanation of the method are available at https://github.com/molgenis/PICALO. A more detailed description of the PICALO method is outlined in the “Methods” section.

We applied PICALO to bulk RNA-seq eQTL datasets in the blood (BIOS [18]; Fig. 1A) and brain (MetaBrain [7]; Fig. 1A). In short, the blood dataset is a large-scale effort from various biobanks in the Netherlands, containing genotype and gene expression data from peripheral blood of population-based samples. The brain dataset is a large-scale meta-analysis of previously published human brain datasets, including multiple brain regions. In the brain, we confined ourselves to the cortex samples from European ancestry. We performed a strict sample selection filter (see the “Methods” section), to exclude any outlier samples, resulting in the inclusion of 2932 samples in the blood and 2440 samples in the brain. We downloaded the summary statistics for the primary eQTLs of each respective study. After filtering, 13,010 eQTLs remained in the blood and 12,080 eQTLs in the brain.

**Fig. 2:**
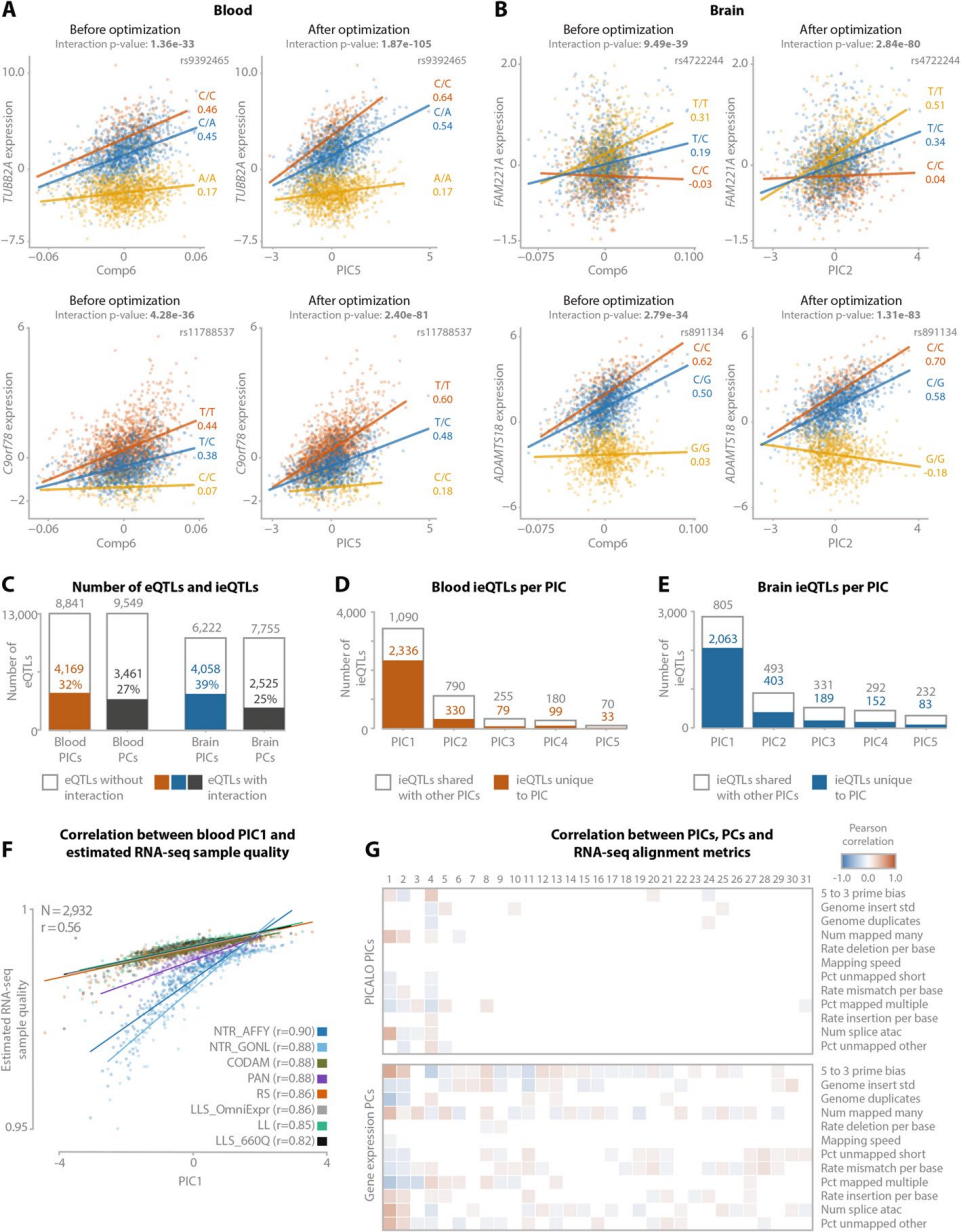
Examples of PICALO optimization for ieQTLs. **A** ieQTL for the genes *TUBB2A* and *C9orf78* with expression PC6 before optimization and resulting PIC5 after optimization in blood. **B** ieQTL for the genes *FAM221A* and *ADAMTS18* with expression PC6 before optimization and resulting PIC2 after optimization in the brain. **C** The number of eQTLs tested in the blood and brain and the respective number of eQTLs that have an interaction with one or more PICs or expression PCs. **D** The number of ieQTLs for the first five PICs in the blood. **E** The number of ieQTLs for the first five PICs in the brain. **F** Regression plot showing the correlation between PIC1 and estimated RNA-seq sample quality calculated as the per-sample expression correlation with the overall average expression. **G** Pearson correlation heatmaps correlating PIC (top) and expression PC (bottom) to RNA-seq alignment metrics in the blood. The correlation *p*-values are corrected for multiple testing with Benjamini-Hochberg, and only correlations with an FDR < 0.05 are shown. Note that many of the expression PCs correlate significantly with RNA-seq alignment metrics while only a limited number of PICs show a significant correlation

We first log_2_ transformed the uncentered gene expression data and adjusted the expression data for known technical covariates such as sex, genotype multidimensional scaling (MDS) components, and dataset indicator variables while retaining the mean and standard deviation. Since we observed high correlations between RNA-seq alignment metrics and measured cell type proportions (Additional file 1: Fig. S1), we did not correct for these metrics as this would remove part of the cell type signal. Moreover, by not explicitly correcting for alignment metrics, we could evaluate PICALO’s performance to distinguish between technical and biological effects. We used the first 25 PCs over this matrix as starting positions for the EM algorithm representing our initial guesses of the eQTL context. After applying PICALO, we observed a large increase in the significance of the interaction term for a large proportion of ieQTLs, for instance, for *TUBB2A* and *C9orf78* (− log_10_ *p*-value increase 71.9 and 45.3) in the blood (Fig. 2A) and *FAM221A* and *ADAMTS18* (− log_10_ *p*-value increase 41.5 and 49.3) in the brain (Fig. 2B). We identified 31 PICs in the blood having a total of 5894 significant interactions (BH-FDR < 0.05) with 4169 unique eQTLs (32%; Fig. 2C; Additional file 1: Fig. S2A; Additional file 2: Table S1; Additional file 3: Table S2). In the brain, we identified 21 PICs having a total of 5481 interactions with 4058 unique eQTLs (39%; Fig. 2D; Additional file 1: Fig. S2B; Additional file 2: Table S1; Additional file 3: Table S3). Each PIC showed little to no correlation with other PICs (Pearson *r* < 0.07 for the blood and < 0.04 for the brain; Additional file 1: Fig. S3) and the majority of ieQTLs interacted with only one PIC (72% in the blood and 75% in the brain), suggesting PICs capture unique effects. The first five PICs correlated moderately with the starting positions (average Pearson *r* = 0.64 ± 0.13 for the blood and 0.51 ± 0.08 for the brain; Additional file 1: Fig. S4), while subsequent PICs showed lower correlations (average Pearson *r* = 0.20 ± 0.15 for the blood and 0.17 ± 0.1 for the brain; Additional file 1: Fig. S4).

### PICs are robust and replicate better than expression PCs

In order to evaluate to what extent PICALO is able to reconstruct hidden covariates, we performed a simulation study. Using the effect sizes as observed in the real data, we simulated expression data containing three hidden contexts. PICALO is able to robustly and efficiently identify simulated interaction context even if the starting vector has minimal correlation with the actual context (*r* ~ 0.2; Additional file 5: Supplementary note). However, PICALO’s reconstruction performance decreases if the starting vector does not interact with a sufficient amount of ieQTLs (Additional file 1: Fig. S5; Additional file 5: Supplementary note). Moreover, we down-sampled both the brain and blood eQTL datasets and observed that the power to detect ieQTLs is dependent on factors such as sample, the number of eQTLs affected by specific contexts, and the overall effect sizes of these interactions (Additional file 1: Fig. S6). Since EM algorithms can yield results that depend on the choice of the initial starting position (i.e., expectation), we evaluated to what extent this was the case for PICALO. We re-optimized the first five PICs in blood using each of the 25 expression PCs as independent starting positions and compared the results (Additional file 1: Fig. S7; Additional file 5: Supplementary note). We observed that PICs capture unique effects that can be robustly identified using PICALO, but that the order in which PICs are identified can be dependent on the starting position. We then further assessed the robustness of the identified PICs in the blood and brain by splitting each expression matrix into genes that map to odd chromosomes (discovery) and genes that map to even chromosomes (replication). We then identified expression PCs and PICs using the odd chromosome (eQTL) genes and subsequently determined how many ieQTLs could be identified with those components on the even chromosome eQTLs. Generally, we observed that PICs with at least 40 ieQTLs replicated in the odd chromosome eQTLs. In the blood, we identified 6 PICs with at least 40 ieQTLs in the odd chromosome eQTLs with which 1658 unique even chromosome eQTLs showed a significant interaction (31%). Comparatively, the same number of expression PCs interacted with 1552 unique even chromosome eQTLs. In the brain, the difference was even larger with 1376 unique even chromosome eQTLs showing a significant interaction compared to 1105 for the same number of expression PCs. Most notably, PICs show a higher specificity than expression PCs by only interacting with one gene in 82% of cases compared to 74% for expression PCs in the blood and 88% compared to 61% in the brain (Additional file 1: Fig. S8).

### PICs capture more interaction variance using fewer components than PCA

We then compared the PIC ieQTLs with expression PC ieQTLs (i.e., the starting position on which PICs are identified) to evaluate to what extent applying PICALO on top of PCA improves in the inference of eQTL context. Using an equal number of expression PCs as the number of identified PICs, we found that PICs interact with a higher proportion of unique eQTLs than expression PCs (5.4% increase in blood, 14.9% increase in brain; Fig. 2C). We then evaluated to what extent the variance explained by the interaction terms of the ieQTL models (i.e., interaction variance) was captured by the identified PICs. For this, we adjusted the expression data for the PICs and their interactions and evaluated if we could detect any ieQTLs on the residuals using the first 25 expression PCs (i.e., the starting positions with which the PICs were optimized). We observed only a limited number of ieQTLs (7 in the blood, 4 in the brain), indicating that the majority of interaction variance that is captured by the first 25 PCs is also captured by PICs. We extended this analysis by also evaluating the first 100 PCs and observed an additional 239 ieQTLs in the blood and 40 in the brain. To confirm that more PICs can be identified at the cost of higher computational complexity, we reperformed PICALO while doubling the number of starting vectors to 50 and reducing the minimum number of EM-iterations to 15. This resulted in 10 additional PICs, each interacting with < 20 eQTLs in the blood and no additional PICs in the brain. The addition of extra starting vectors did not influence any of the first 21 PICs. Together, these results suggest that the number of PICs is dependent on the number of starting vectors supplied to PICALO. However, additional starting positions only marginally increase the total number of ieQTLs, while increasing the computational cost substantially.

### RNA-seq quality and technical confounders are major drivers of eQTL effect sizes in the blood and brain

We have shown that PICs are robust proxies for contexts that affect eQTL effect sizes. However, since PICs lack specific annotation, further analysis is required for interpretation. To provide these annotations, we correlated the PICs to known technical factors (e.g., estimated RNA-seq sample quality, RNA-seq alignment metrics, sample annotations; Additional file 6: Table S4 and Additional file 7: Table S5) and biological phenotypes (e.g., measured or predicted cell type proportions; Additional file 6: Table S4 and Additional file 7: Table S5). Subsequently, we evaluated single-cell expression of genes that highly correlate with PICs to further investigate cell type enrichments. Finally, we performed gene set enrichment analysis to investigate pathway and cell type enrichment of positively and negatively correlated genes using the ToppGene suite [26]. Analogous to other types of hidden variables, PICs may capture the influence of multiple distinct biological and technical factors. Like those other methods, factors with a large effect on eQTLs can be the dominant source of variance within a single component, allowing for the classification of PICs into predominantly technical and non-technical contexts. This enables the correction of technical influences while retaining informative biological variance for downstream analyses.

The PIC1 that was identified in the blood and the PIC1 identified in the brain individually interact with a substantial number of eQTLs (3426 in the blood and 2868 in the brain; Fig. 2D, E). Upon further analysis, both PIC1s showed a high correlation with estimated RNA-seq sample quality (blood Pearson *r* = 0.56, Fig. 2F; brain Pearson *r* = − 0.66, Additional file 1: Fig. S9B) that we calculated per sample as the correlation between the gene expression per gene and the average expression per gene over all samples. Notably, in the blood, the correlation per cohort was substantially higher (Pearson *r* > 0.82) than the joint correlation (Pearson *r* = 0.56), suggesting heterogeneity of the estimated RNA quality and consequently that PIC1 provides a more reliable quality measure, perhaps by capturing multiple aspects of technical variation in a single component. We further observed that 88% of eQTLs that interact with PIC1 have a positive interaction effect, suggesting an increased effect size in high-quality samples, both in the blood and in the brain. Since we did not correct the expression data for RNA-seq quality, it makes sense that PIC1 reflects this.

We then determined how many PICs were affected by RNA-seq alignment metrics and compared this to expression PCs. We calculated the Pearson correlations between each PIC and RNA-seq alignment metrics (e.g., those from PICARD, FastQC; Fig. 2G; Additional file 1: Fig. S10) and observed that 10 out of 31 PICs had a significant PIC-metric correlation in the blood (9.4% of pairs; BH-FDR < 0.05) and 7 out of 21 PICs in the brain (16.1% of pairs). In contrast, for an equal number of expression PCs, we observed that 29 out of 31 expression PCs had a significant correlation in the blood (41.7% of pairs) and 21 out of 21 expression PCs in the brain (49% of pairs). This indicates that PICs distinguish technical from non-technical factors using fewer components than PCs and that most PICs do not reflect known technical confounders. We further observed that PIC1 shows significant interaction effects for many more eQTLs, as compared to the number of significant interaction effects for expression PC1, both in the blood and in the brain. This indicates that while RNA quality (captured by both PIC1 and PC1) has a substantial effect on eQTL effect sizes, PIC1 reveals many more eQTLs that have a significant interaction effect with RNA quality.

**Fig. 3:**
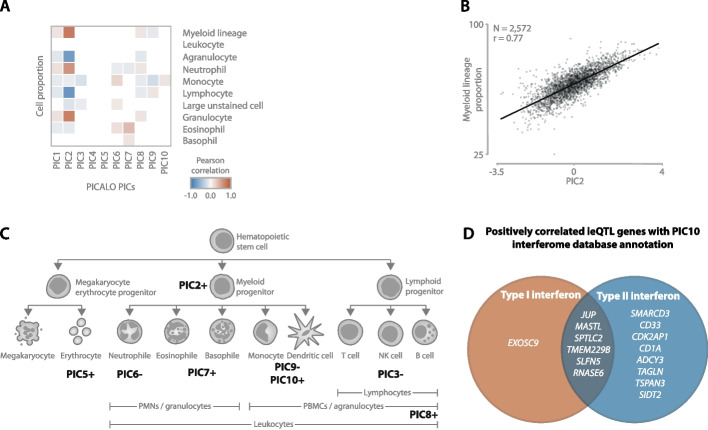
**A** Pearson correlation heatmap correlating PICs to measured cell type proportions in the blood. The correlation *p*-values are corrected for multiple testing with Benjamini-Hochberg, and only correlations with an FDR < 0.05 are shown. **B** Regression plot showing the correlation between PIC2 and myeloid lineage cell proportions (granulocyte + monocyte) in the blood. **C** Simplified overview of the blood cell type lineage with annotations of PICs describing distinct (groups of) cell types using measured cell type proportions, gene set enrichments, and single-cell expression enrichment. Positive and negative signs indicate the direction of the effect. Only the first 10 PICs are considered. An image of the top layer cell type is created with BioRender.com. **D** Negatively correlating eQTL genes interacting with PIC10 showed enrichment for type II interferon signaling as annotated by the Interferome Database Annotation

**Fig. 4:**
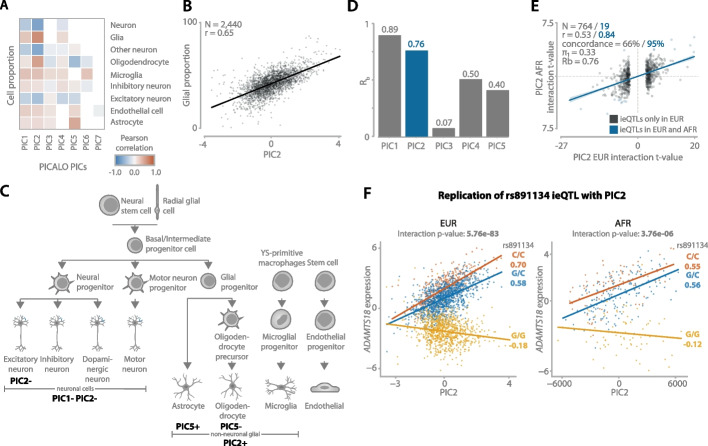
**A** Pearson correlation heatmap correlating PICs to predicted cell type proportions in the brain. The correlation *p*-values are corrected for multiple testing with Benjamini-Hochberg, and only correlations with an FDR < 0.05 are shown. **B** Regression plot showing the correlation between PIC2 and glial proportion (microglia + oligodendrocyte + astrocyte) in the brain. **C** Simplified overview of the brain cell type lineage with annotations of PICs describing distinct (groups of) cell types using predicted cell type proportions, gene set enrichments, and single-nucleus expression enrichment and replication. Positive and negative signs indicate the direction of the effect. Only the first seven PICs are considered. Images of the bottom layer cell types are created with BioRender.com. **D** *R*_*b*_ replication statistics for the replication of eQTLs interacting with the first five PICs discovered in samples of European ancestry (EUR) and replicated in samples of African ancestry (AFR). **E** Regression plot showing the interaction *t*-values of PIC2 ieQTLs discovered in EUR and replicated in AFR. Blue points are significant in both datasets, the statistics of which are shown in blue. The shaded area indicates the 95% confidence interval. **F** Example of replicating ieQTL: rs891134 affecting *ADAMTS18* gene expression and interacting with PIC2. The left plot shows the interaction in EUR, and the right plot shows AFR. The *x*-axis shows the PIC2 scores, the *y*-axis shows the covariate corrected gene expression, and each dot represents a sample. The *p*-values are calculated using the unconditional ieQTL analysis. Colors indicate SNP genotype. Values under the alleles are Pearson correlation coefficients

We next classified PICs as being predominantly technical if they were correlated significantly with an RNA-seq alignment metric, but not with one of the cell-count proportions or other biological factors. Using this principle, we determined that PIC1, PIC4, and PIC8 are predominantly technical in the blood (Figs. 2G and 3A) and PIC1, PIC4, and PIC7 in the brain (Additional file 1: Fig. S10A; Fig. 4A). In total, the technical PICs accounted for 3760 interactions (64%) with 3555 unique eQTLs (27%) in the blood and 3416 interactions (62%) with 3140 unique eQTLs (31%) in the brain. The remaining non-technical PICs accounted for 2134 interactions (36%) with 1617 unique eQTLs (12%) in the blood and 2065 interactions (38%) with 1671 unique eQTLs (16%) in the brain. In the blood and brain combined, we identified non-technical interaction effects for 3065 unique genes.

For the interpretation of the non-technical PICs, we confined ourselves to those PICs with a minimum of 40 ieQTLs based on our simulation analyses (first 10 PICs in the blood, first 7 in the brain). We note however that true eQTL context may interact with only a handful of genes, and therefore, highly informative biological context might be overlooked (Additional file 5: Supplementary Note).

### Specific types of cells, interferon signaling, and prior cytomegalovirus infection influence eQTL effect size in the blood

To interpret the non-technical PICs in the blood, we first correlated their values with measured cell counts and combinations of cell types from the same lineage (Fig. 3A; Additional file 1: Fig. S11A). For 7 out of 10 PICs, we observed a significant correlation (PIC1-PIC3, PIC6-PIC9; BH-FDR < 0.05). For example, PIC2, interacting with 1120 eQTLs, showed the strongest correlation with myeloid lineage cell percentage (Pearson *r* = 0.77; Fig. 3B), consisting predominantly of neutrophils. Furthermore, for PIC7, interacting with 60 eQTLs, we observed a correlation with measured eosinophil proportion (Pearson *r* = 0.36).

We then evaluated whether PICs were enriched for certain cell types and biological processes by correlating the PICs with gene expression levels and splitting the genes into groups of positively and negatively correlating ones. Using the top 200 positively and 200 negatively correlated genes per PIC (Additional file 8: Table S6), we tested for gene set enrichments (Additional file 9: Table S7) and evaluated expression in purified and single-cell datasets (Additional file 1: Fig. S12 and Fig. S13). This analysis further supported the neutrophil association for PIC2 and the eosinophil association for PIC7. Furthermore, we were able to assign putative labels of (sub)types of cells to 6 more PICs (Fig. 3C). For PIC5 (103 ieQTLs), for example, we found that the positively correlating genes showed a strong enrichment of erythrocyte-specific genes (ToppCell enrichment *p*-value < 1.4 × 10^−233^). Moreover, this PIC was enriched for red blood cell pathways, such as for the uptake of oxygen and release of carbon dioxide (pathway enrichment *p*-value = 6.7 × 10^−11^), among others (Additional file 9: Table S7). The results for PICs with < 40 ieQTLs but with clear annotations can be found in Additional file 5: Supplementary note.

Next, we evaluated if the top 200 positively and negatively correlated genes per PIC were enriched for certain pathways. In the Zhernakova et al. study [18], which uses the same data as this study, an interaction module was identified that was a proxy for type 1 interferon response. Here, we found that PIC10 was enriched for the interferon signaling pathway (pathway enrichment *p*-value = 1.6 × 10^−36^). While the number of affected eQTLs by PIC10 was lower than the number of eQTLs affected by the equivalent module in Zhernakova et al. (44 as compared to 145), the enrichment *p*-value for the interferon signaling pathway was substantially more significant (*p*-value = 1.6 × 10^−36^ versus *p*-value = 2 × 10^−6^). Finally, by using the interferome database annotation [27], we found that 21 out of 22 genes that positively correlate with PIC10 were involved in type 2 interferon signaling (Fig. 3D).

Finally, we found a significant correlation (Pearson *r* = − 0.29, *p*-value = 5.97 × 10^−15^; Additional file 1: Fig. S14A-B) between PIC3 and the presence of antibodies against cytomegalovirus (CMV). This was determined by a CMV infection signal that was calculated from an aggregate of multiple IgG antibody profiles enriched in a PhIP-seq experiment for 1433 samples [28]. These CMV antibodies can be indicative of a past or latent infection with CMV, which is expected for 45% of the Dutch population [29]. Latent CMV infection can have a lasting effect on the immune system [30] and increases monocyte differentiation [31], and CMV can reprogram monocyte gene expression [32, 33]. We also observed a correlation between the CMV antibodies and expression PC5 (*r* = − 0.29, *p*-value = 1.14 × 10^−15^). Although PIC3 and PC5 seem very similar at first glance (Additional file 1: Fig. S14C; Pearson *r* = 0.66), we observed that PC5 was correlated with multiple cell type proportions (maximum absolute Pearson *r* = 0.27 with neutrophil proportion; Additional file 1: Fig. S14D), while PIC3 was not (maximum absolute Pearson *r* = 0.14 with monocyte proportion; Additional file 1: Fig. S14D). This difference emphasizes how PICs, unlike expression PCs, can disentangle the influence of CMV infection on eQTLs into separate components, either affecting cell type proportions or gene regulation.

### Specific types of cells influence eQTL effect size in the brain

In contrast to the PICs in the blood, the PICs identified in the brain were harder to annotate since no measured cell type proportion measurements were available for this dataset. However, by using the predicted cell type proportions as used in de Klein et al. [7] (Additional file 1: Fig. S11B), gene set or pathway enrichment analysis (Additional file 9: Table S7), a comparison with brain single-cell expression from ROSMAP (Additional file 1: Fig. S13B), a comparison with cell type ieQTLs by de Klein et al. [7], and finally a replication using the single-nucleus based eQTL dataset by Bryois et al. [34] (Additional file 1: Fig. S15 and Additional file 1: Fig. S16), we were able to assign putative labels to a total of three out of seven PICs.

In order to determine the replication of PIC ieQTLs, we evaluated different aspects of replication: allelic concordance (AC), *R*_*b*_ [35], and *π*_1_ [36]. AC is an indication of the proportion of effects that have a shared direction and are significant in both discovery and replication dataset, *R*_*b*_ estimates the correlation between effect slopes while controlling for potential covariance in standard errors of those slopes, and *π*_1_ estimates the proportion of effects that are true positive in the replication cohort but does not take into account effect direction and can be dependent on replication dataset sample size.

First, PIC2, which has a significant interaction with 896 eQTLs, correlated strongly with predicted glia proportion (Pearson *r* = 0.65). Moreover, PIC2 showed a high replication with single-nucleus eQTLs from oligodendrocytes (AC = 88%, *R*_*b*_ = 0.83, *π*_1_ = 0.87; Additional file 1: Fig. S15 and Fig. S16), the most common glial cell, further supporting this association. Second, for PIC5, we identified 315 ieQTLs and strong correlations with predicted astrocyte (Pearson *r* = 0.48) and oligodendrocyte proportion (Pearson *r* = − 0.45) as well as a high enrichment of cell type-specific genes for these cell types (*p*-value < 1.5 × 10^−136^ for astrocyte and *p*-value < 6.2 × 10^−99^ for oligodendrocyte). This association is further supported by a high replication rate with astrocyte sn-eQTLs (AC = 76%, *R*_*b*_ = 0.67, *π*_1_ = 0.58; Additional file 1: Figs. S15 and S16). The results for PICs with < 40 ieQTLs but with clear annotations can be found in Additional file 5: Supplementary note.

### Top brain PICs replicate well across datasets

Lastly, we evaluated how well PIC ieQTLs replicate across datasets. For this, we used the PICs identified in the 2440 brain samples from European ancestry (EUR) and replicated them in 311 brain samples from African ancestry (AFR). Given that the AFR samples originate from cohorts that also include EUR samples, we expect to replicate both biological as well as technical contexts in the AFR samples. We observed that the top two PICs replicate very well (PIC1: AC = 76%, *R*_*b*_ = 0.89, *π*_1_ = 0.3; PIC2: AC = 66%, *R*_*b*_ = 0.76, *π*_1_ = 0.33; Fig. 4D, E; Additional file 1: Fig. S17; Additional file 10: Table S8). The high concordance of technical PIC1 is most likely due to the overlap of shared confounding effects between EUR and AFR samples originating from the same cohorts. As expected, the remaining PICs replicate less well as the number of ieQTLs decreases with subsequent PICs, and they capture a lower proportion of interaction variance. For the biological PIC2, annotated as glial cell proportion, a total of 10 ieQTLs were replicated. This included *ADAMTS18* (Fig. 4F), which has previously been identified as oligodendrocyte-specific [37]. This finding supports our earlier observation that PIC2 captures glial proportion differences.

## Discussion

In this manuscript, we have highlighted a possible confounding effect of technical variation on the detected interaction eQTLs. Although RNA-seq quality metrics or expression PCs are usually considered as confounders, their interactions with genotype are, to our knowledge, never accounted for. However, such effects could be observed when samples with lower quality have increased error margins in their measured expression compared to samples with higher quality. For instance, when the samples would be stratified on RNA quality, this could result in different observed eQTL effect sizes, because of the differences in eQTL detection power between high-quality and low-quality samples, in turn resulting in an interaction effect.

Overall, the purpose of PICALO is to identify hidden contexts that mediate eQTL effect sizes. As reported before [2], we have shown that cell type proportion is an important biological context to consider. However, although eQTLs are plentiful, our results suggest that a modest proportion of eQTLs identified in bulk data are context-specific for biological contexts. This could be because cell type regulatory effects can be shared between cell types from the same lineage or because they could be dependent on extracellular stimulation. We therefore underline that even after PICALO optimization, we expect the number of true interaction eQTLs to be limited to a small set of genes.

PICALO has a number of limitations. Like any hidden variable inference method, the identified PICs do not have a direct functional annotation and therefore require further analyses for proper interpretation. While we have shown that this is feasible, we acknowledge that the confidence in the annotation is dependent on the quality and availability of ground truth information such as gene pathways, cell type specificity of genes, and phenotypic and technical covariates in the dataset. Furthermore, while we have shown that PICs are independent to each other and capture distinct effects, as well as are able to disentangle technical from biological effects, it is still possible that PICs capture a mixture of contexts which may complicate the interpretation. As a result, the interpretation of PICs, interacting with only a small set of eQTLs, should be done with caution. Moreover, the performance of PICALO is dependent on factors such as sample size, the number of eQTLs affected by specific contexts, and the overall effect sizes of these interactions. We envision that sample sizes of molecular QTL datasets will increase substantially in the near future. PICALO is particularly well suited to such larger datasets, because it will benefit from having more significant ieQTLs to start optimization with, resulting in improved power to increase the number and accuracy of identified hidden contexts. Finally, we note that PICs are currently restricted to additive, linear effects and that lowly expressed genes cannot be included as their interactions cannot be properly corrected for.

## Conclusions

We have developed PICALO, a hidden variable inference method that applies an EM algorithm to identify hidden contexts that affect eQTL effect sizes. We have applied it to a large blood and brain bulk eQTL dataset and identified a considerable number of contexts and ieQTLs, reflecting thousands of genes, which is a substantial improvement over Zhernakova et al. who reported significant interactions for only 12% of the eQTLs [18]. While we could also assign a biological label to thousands of ieQTLs, many of the identified eQTLs interacted with technical PICs. This could suggest that for a large proportion of eQTLs, the effect size depends on technical covariates even after stringent correction. We therefore reason that the technical PICs identified by PICALO can be used to remove additional technical variance from eQTL studies to further improve eQTL effect size estimates, which in turn might improve downstream analyses with GWAS signals such as colocalization or Mendelian randomization. We demonstrated that PICALO outperforms PCA by capturing this technical effect within a limited number of components and showing better replication of the ieQTLs. Overall, the identified PICs describe highly relevant biological and technical eQTL contexts without knowing them a priori. Moreover, they provide better differentiation between technical and biological influences, while using fewer components as compared to PCA.

Application of PICALO is not limited to bulk RNA-seq and could also be applied in single-cell data to identify stimulation contexts and specific types of cells including the less frequent ones. We have shown that expression PCs can be used as a starting position for PICALO (i.e., the initial guess of eQTL context), but we do note that it is also possible to use marker gene expression levels or cell-type proportions. We expect that PICALO can also be applied to other quantitative phenotypes, such as clinical data, ancestry information, or other molecular phenotypes such as protein levels. While we have focused on *cis*-eQTLs in this study, PICALO could also be applicable to *trans*-eQTLs. For many *trans*-eQTLs, it is currently unclear whether they are the consequence of cell type proportion differences or actual regulatory effects [38], a distinction that can potentially be improved using a combination of PICs identified by *PICALO*.

To sum up, PICALO is insightful for the analysis of eQTL datasets to detect the relevant contexts that influence eQTLs. Currently, our method is especially well-powered in large sample sizes, but various large-scale eQTL and pQTL studies are currently underway or have just been completed [38, 39]. Therefore, we believe PICALO will prove highly useful in the future, enabling the discovery of previously unknown contexts that influence eQTL effect sizes and consequently potentially improving the interpretation of disease-associated variants.

## Methods

### General description of PICALO

PICALO is an EM-based algorithm for the identification of known and unknown contexts that influence the regulatory effect of genetic variants on gene expression. PICALO takes as input a set of eQTLs and their corresponding expression and genotype data, as well as a set of starting positions (e.g., potential context components). Optional arguments allow for the correction of technical covariates both with and without an interaction term. PICALO can deal with missing genotypes and can work with multiple, heterogeneous eQTL datasets. In this paper, we show the use of expression PCs as a starting position; however, other quantitative phenotypes or characteristics such as cell type fractions or marker genes can also be used. Using this data, PICALO identifies hidden variables that maximally affect eQTL effect sizes (i.e., PICs) in three steps. These steps are detailed below.

#### Step 1: eQTL inclusion criteria and data pre-processing

PICALO allows for the filtering of eQTLs based on the following metrics: eQTL *p*-value (default < 0.05), genotype call rate (default > 0.95), number of samples per allele (default > 2), minor allele frequency (MAF; default > 0.05), and Hardy–Weinberg equilibrium *p*-value (default > 1 × 10^−4^). If the input data consists of multiple datasets, the call rate is calculated per dataset and all samples of the same dataset are considered missing if the call rate is not met. Note that eQTLs for which the included samples have a low average expression (e.g., log_2_ (trimmed mean of *M* values; TMM + 1) < 1) should be manually removed prior to applying PICALO.

#### Step 2: correcting the gene expression levels for technical confounders

For the gene expression, PICALO expects the input to be log_2_-transformed, centered per gene, and *z*-transformed per sample over all genes. To correct for technical confounders, PICALO constructs a design matrix encompassing all supplied technical covariates and automatically includes dataset indicator variables if more than one dataset is used. An extra term for the interaction between a technical covariate and genotype can be included as well. If applicable, dataset indicator variables always include a term for the interaction with genotype to correct for possible dataset-specific interactions. Before the identification of each PIC, the input gene expression data is corrected for all terms in the design matrix using OLS regression in which samples with missing genotypes are ignored and remain missing. The residuals are used as gene expression input for step 3.1. After identification, each PIC is included in the design matrix with a term for the interaction with the genotype.

#### Step 3.1: expectation—identification of interaction eQTLs

As the first part of the EM step, PICALO identifies the starting position that has the highest number of significant ieQTLs. A graphical overview of this step is shown in Additional file 1: Fig. S19A. The starting position is an initial guess of the eQTL context and is hereafter referred to as context. The significance of an ieQTL is analyzed as follows: first, the genotype and context effects are regressed out from the expression data, ensuring that only the interaction term explains any variance in the model. PICALO then forces the distribution of the gene expression levels and the context(s) into a normal distribution per dataset by ranking with ties to prevent the influence of outliers. Per eQTL, the significance of the interaction term is calculated by comparing the residual sum of squares (RSS) of two linear models: one without (Eq. 1) and one with (Eq. 2) the interaction term included.

1 $$y= {\beta }_{1}+{\beta }_{g}\bullet g+{\beta }_{c}\bullet c+ \varepsilon$$

2 $$y= {\beta }_{1}+{\beta }_{g}\bullet g+{\beta }_{c}\bullet c+ {\beta }_{gxc}\bullet g\bullet c+ \varepsilon$$

*where*

$$y=\text{gene expression}; \beta ={\text{beta}};g={\text{genotype}};c={\text{context}}$$

$$\varepsilon ={\text{residuals}}; gxc=\mathrm{genotype\ context\ interaction}$$

The interaction *p*-value is calculated using a one-sided *F*-statistic with (1, *n* − 4) degrees of freedom after which BH multiple testing correction is applied per context. Only ieQTLs that are significant (BH-FDR < 0.05) are used for the optimization step. If there are less than two ieQTLs available for optimization for any of the samples, the program is halted.

#### Step 3.2: Maximization — optimization of interaction component

For the optimization of the context, PICALO maximizes the log-likelihood of the interaction model (Eq. 3) over all significant ieQTLs considering each sample individually. A graphical overview of this step is shown in Additional file 1: Fig. S19B. For clarity, first, consider the case in which only one ieQTL is optimized. Using the beta estimates as calculated in Eq. 2, the log-likelihood of these beta values, given the observations (e.g., gene expression levels, genotype, context), can be calculated as follows:

3 $$\begin{array}{c}\begin{array}{c}L({\beta }_{1},{\beta }_{g},{\beta }_{c},{\beta }_{gxc},{s}^{2})=\\ {\text{log}}\prod\limits_{i=1}^{n}p\left({y}_{i}|{g}_{i},{c}_{i};{\beta }_{1},{\beta }_{g},{\beta }_{c},{\beta }_{{\text{gxc}}},{s}^{2}\right)=\\ \sum\limits_{i=1}^{n}{\text{log}}p\left({y}_{i}|{g}_{i},{c}_{i};{\beta }_{1},{\beta }_{g},{\beta }_{c},{\beta }_{{\text{gxc}}},{s}^{2}\right)=\end{array}\\ -\frac{n}{2}{\text{log}}2\pi -n{\text{log}}s-\frac{1}{2{s}^{2}}\sum\limits_{i=1}^{n}\left({y}_{i}-\left({\beta }_{1}+{\beta }_{g}\bullet {g}_{i}+{\beta }_{c}\bullet {c}_{i}+ {\beta }_{gxc}\bullet {g}_{i}\bullet {c}_{i}\right)\right)\end{array}$$

where

$$L={\text{likelihood}}; \beta ={\text{beta}};s=\text{standard deviation};n= \text{sample size};$$

$$p={\text{probability}};y=\text{gene expression}; g={\text{genotype}};c={\text{context}}$$

$$gxc=\text{genotype context interaction}$$

In other words, the likelihood of the model equals the joint probability of the model residuals coming from a normal distribution with a mean 0 and a standard deviation of ${s}^{2}$. In contrast to how the likelihood is traditionally applied (i.e., likelihood of model parameters given the data), PICALO calculates the likelihood of the context value for a single sample given the model parameters. The underlying assumption is that the context estimate of a given sample has a certain margin of error but that this error has an average of 0 over all samples (i.e., the model parameters are error-free). PICALO calculates the log-likelihood of ${{\text{sample}}}_{i}$ having context value ${c}_{i}$ given that ${\beta }_{c}$ and ${\beta }_{gxc}$ are true. In other words, PICALO adjusts the value for ${c}_{i}$ to maximize the log-likelihood of the complete model while all other observations and parameters, especially all values for $c\ne {c}_{i}$, are kept fixed. In the case of a single ieQTL, this translates to adjusting the context value of each sample (Additional file 1: Fig. S20A) and determining the change in log-likelihood (Additional file 1: Fig. S20B). In principle, the log-likelihood is maximal for a given sample when its context value intersects with the regression line of its corresponding genotype group.

We assume ${s}^{2}$ to be constant since we forced the expression and context distributions into a normal distribution per dataset by ranking with ties (see step 3.1). As a result, maximizing the log-likelihood equals minimizing the RSS of the model (Eq. 4).

4 $${\sum }_{i=1}^{n}\left({y}_{i}-\left({\beta }_{i}+{\beta }_{g}\bullet {g}_{i}+{\beta }_{c}\bullet {c}_{i}+ {\beta }_{gxc}\bullet {g}_{i}\bullet {c}_{i}\right)\right)$$

where

$$y=\text{gene expression}; \beta ={\text{beta}};g={\text{genotype}};c={\text{context}}$$

$$gxc=\text{genotype context interaction}$$

Extending this concept to $m$ ieQTL models, we can find the joint maximum log-likelihood by identifying the context value that gives the lowest RSS over all ieQTLs. This optimum can be calculated efficiently by describing the log-likelihood per sample and per model as coefficients of a second-degree polynomial, summing these together per sample, and finally identifying the focus of the joint function (Additional file 1: Fig. S20B).

We then repeat this process by using the optimized context as input context at the start of step 3.1. Note that in the second round, the optimized context is always used as the starting context for optimization; therefore, the starting position selection step is skipped. If the minimum number of iterations has been reached (default 5), and the Pearson correlation between the current and previous optimized context vectors is above the tolerance (default ≥ 0.999), the covariate is considered converged and the loop is terminated. This converged covariate is referred to as a PIC. In some cases, the optimization step can get stuck in an oscillating loop in which the values of the context in subsequent iterations are reverted to those of previous ones. If this occurs, the context is also considered converged and the iteration (e.g., current or previous) with the highest number of ieQTLs is returned as PIC. Steps 2 and 3 are repeated until the required number of PICs is identified or < 2 ieQTLs are available for optimization. Note that a specific starting position can be used more than once to derive a PIC. As a result, the number of PICs that PICALO identifies is not limited to the number of starting positions that are supplied.

### Data pre-processing

We made use of the bulk RNA-seq eQTL datasets collected by BIOS [18] (peripheral blood; *n* = 3997) and MetaBrain [7] (multiple brain regions; *n* = 8727). For BIOS, we confined ourselves to the samples included in eQTLgen (*n*-samples = 3831, *n*-cohorts = 9) [38] and used the RNA-seq data as aligned and pre-processed for that project. For MetaBrain, we confined ourselves to the cortex samples of European ancestry (EUR; *n*-samples = 2683, *n*-cohorts = 14) and African ancestry (AFR; *n*-samples = 319, *n*-cohorts = 3). The EUR samples were used for discovery and the AFR samples for replication and were processed independently. In short, all samples were realigned in the same manner, and the genotyping array data was imputed using the Haplotype Reference Consortium (HRC) panel. Details on the quality control can be found in the respective manuscripts.

### Genotype data processing

We first ran MixupMapper [40] to identify sample mix-ups in BIOS. In short, MixupMapper identifies *cis*-eQTLs on a dataset and determines if samples are more often an outlier than could be expected by chance. This resulted in 15 possible sample mix-ups that were excluded from the BIOS dataset. For MetaBrain EUR, we removed 223 genetically similar individuals (pihat > 0.125) as calculated with PLINK 2.0 [41]. Furthermore, we excluded all samples for which RNA-seq alignment metrics or sex information was unavailable, as well as datasets with less than 30 samples. Over the remaining samples, we determined the first four MDS components over the genotype data using PLINK. We included variants that passed the following thresholds: MAF > 0.1, Hardy–Weinberg exact test *p*-value > 0.001, and missingness per variant < 5%. We then pruned the SNPs using the independent pairwise setting with a window size of 1000 kb, step size of 50, and ${R}^{2}$ threshold of 0.5. In total, 289,059 variants for BIOS and 52,635 variants for MetaBrain EUR passed pruning and were used to identify the first four MDS components (Additional file 1: Fig. S21). In BIOS, we then excluded a total of 20 samples that had a *z*-score > 3 standard deviations on one of the four components. For MetaBrain EUR, we observed that European Nucleotide Archive (ENA) samples formed a separate cluster (Additional file 1: Fig. S22), most likely because these samples have RNA-seq-derived genotypes. While these genotypes are reliable and can be used for local imputation and eQTL mapping, we found that the non-genome-wide coverage induced batch effects when applying PICALO. We therefore excluded this dataset consisting of 243 individuals. In the MetaBrain AFR samples, we excluded 8 outlier samples. Finally, 2932 samples remained in BIOS, 2440 samples in MetaBrain EUR, and 311 samples in MetaBrain AFR for which we reidentified the genotype MDS components to use as technical confounders (Additional file 1: Fig. S23A and B).

### Initial eQTL context prediction and gene expression pre-processing

We used the TMM-normalized expression data for BIOS and MetaBrain separately. First, we estimated an initial guess of the eQTL context required by PICALO by performing PCA over the uncentered, but covariate-corrected gene expression matrix. This procedure was as follows: we selected the included samples, removed genes with no variation, log_2_-transformed, and saved the mean and standard deviation per gene. As covariates, we used dataset indicator variables, sex, and the four genotype MDS components which we regressed out using OLS regression. We then restored the mean and standard deviation in the residuals, as calculated per gene prior to the covariate correction, and then performed PCA analysis (Additional file 1: Fig. S23C and D). The first 25 expression PCs were saved as starting positions (i.e., expectations) for maximization by PICALO.

We then pre-processed the gene expression matrix to use as input for PICALO. For this, we took the TMM expression data for BIOS and MetaBrain (performed separately) and applied the following steps: we selected the included samples, removed genes with no variation, performed log_2_-transformation, centered and scaled the genes, and *z*-score transformed the samples.

Last, we prepared the gene expression matrix to be used for gene set enrichment analysis. For this, we took the gene expression matrix as prepared above (i.e., TMM log_2_-transformed, centered and scaled, *z*-transformed) and corrected for the included technical covariates (i.e., sex, four genotype MDS components, and dataset indicator variables) from the expression data using OLS regression. To verify that there was no major residual between-datasets variance, we performed PCA and observed that datasets clustered together (Additional file 1: Fig. S23E and F).

### eQTL data processing

We next downloaded eQTLs for BIOS and MetaBrain from molgenis26.gcc.rug.nl and metabrain.nl. We only included primary eQTLs and excluded meta-gene eQTLs. We further removed eQTLs for which the genes had an average expression (TMM log_2_ + 1) < 1 in our sample subset, or for which the SNP had a MAF < 5%, Hardy–Weinberg exact test *p*-value < 0.0001, or those that had less than two samples in each genotype group. This left 13,010 eQTLs for BIOS and 10,280 eQTLs for MetaBrain that we used for downstream analyses.

### PICALO analysis

We determined the optimum number of expression PCs to correct for by testing 0 up to and including 100 expression PCs in steps of 5 and determined the number of expression PCs that maximizes the number of found ieQTLs. This resulted in 40 expression PCs for the blood and 80 for the brain that were included as technical covariates (note that the first 25 PCs of these sets were used as proxies of eQTL context and serve as starting positions for PICALO). We further included dataset indicator variables, sex, and the first four genotype MDS components including their interactions with genotype as technical covariates. We then ran PICALO with a minimum of 50 iterations and a maximum of 100 iterations. We found that forcing a higher minimum number of iterations (default is 5) can in rare situations result into more robust PICs as the program has more time to climb out of local minima. We do note that in the majority of the cases, the model converged within 20 iterations.

### Detection of eQTL interacting with PICs or expression PCs

In order to make a fair comparison between the number of ieQTLs with PICs versus expression PCs, we remapped these interactions separately from PICALO similar to those outlined in the “Methods” section step 1 and step 3.1. However, in this case, we did not remove the genotype and covariate effects from the expression data prior to performing the *F*-test. Moreover, we performed this as a conditional analysis: first, we determined the ieQTLs significant for the first input context (PIC or expression PC). Then, we corrected for the first input context and its interaction with the genotype from the gene expression levels using OLS regression. Finally, we repeated this process for subsequent input contexts, where all previous input contexts were also included in the OLS regression correction. This conditional analysis ensures that the number of ieQTLs is not inflated due to correlated covariates.

### Simulation analysis

To evaluate the performance of PICALO, we tested it using a simulation study using simulated expressed with biologically reliable genotype interactions. We simulated the main eQTL effects for 13,059 *cis*-eQTLs, using a main genotype effect-size and minor allele frequency as we had observed per cis-eQTL in the blood data. For each eQTL, we also modeled a context and interaction effect with the context and main context effect, again as observed using the first three expression PCs forced into a normal distribution per dataset by ranking with ties (i.e., mean = 0, standard deviation = 1) in the blood data. To generate the three simulated contexts, we randomly sampled from a normal distribution with mean 0 and standard deviation 1 (Additional file 1: Fig. S5A and B; Additional file 5: Supplementary note). As a starting vector for PICALO optimization, we simulated three random starting vectors that showed a certain correlation with the simulated contexts, ranging from 0 to 1.0 in increasing steps of 0.1. We then ran PICALO on each set of starting vectors, permitting us to ascertain for each of these starting vectors to what extent PICALO yielded PICs that reflected the simulated contexts. We defined the reconstruction accuracy as the Pearson correlation between the identified PIC and the simulated context. Since the order of PICs is dependent on the effect size of the simulated contexts, we assign each PIC to the context with the highest correlation.

### Detection of ieQTLs for different sample sizes and effect sizes

We evaluated to what extend PICALO is applicable to datasets with different sizes. For this, we randomly selected between 250 and 2500 samples in steps of 250 and applied PICALO using the same set of eQTLs and starting vectors for each subset. We retained the relative contribution of each dataset to the total sample size identical over each subset. In the brain, we excluded *n* = 250 to prevent datasets from being excluded due to too few samples (minimal 30 samples per dataset).

### Replication of ieQTLs within the dataset

We evaluated the robustness of the identified PICs in the blood and brain by splitting each expression matrix into genes that map to odd chromosomes (discovery) and genes that map to even chromosomes (replication). We chose to use the odd chromosomes for discovery since it had a higher number of genes and eQTLs. Moreover, we did not consider genes that mapped to the sex chromosomes. We then identified expression PCs and PICs using the odd chromosome (eQTL) genes and ascertained how many ieQTLs could be identified with those components on the even chromosome eQTLs. We considered ieQTLs with a BH-FDR < 0.05 to be significant.

### Gene set enrichment analysis

In order to annotate the PICs, we performed functional enrichment of cell types and pathways using the ToppGene functional analysis tool [26]. For this, we used the covariate-corrected gene expression matrix and forced the expression distribution per gene into a normal distribution per dataset by ranking with ties. We removed genes that had an average expression (TMM log_2_ + 1) < 1 in our sample subset and calculated the Pearson correlation between each PIC-gene pair. Two sets of gene enrichment analyses were performed: (1) over the top 200 genes with the highest or lowest significant (BH-FDR < 0.05) Pearson correlation *z*-score and (2) over the ieQTL genes. In both cases, the results were split into positively and negatively correlating genes. Standard ToppGene settings were used, and only significant results were reported (FDR < 0.05).

### BLUEPRINT purified cell expression data

We downloaded the BLUEPRINT [42] bulk RNA-seq expression of purified venous blood and cord blood fractions from http://blueprint-data.bsc.es/. In case of multiple experiments on the same cell type, we took the mean fragments per kilobase of transcript per million (FPKM) value per gene. Genes with a mean FPKM smaller than one and that showed no expression in at least 90% of the cell types were excluded. We log_2_ transformed the FPKM + 1 values and performed a center and scaling per sample.

### ROSMAP brain single-cell expression data

We downloaded the ROSMAP [43] single-nucleus RNA-seq data from Synapse. This dataset encompasses 80,660 single-nucleus transcriptomes from the prefrontal cortex of 48 individuals with varying degrees of Alzheimer’s disease pathology. The pre-processing of the data is described in de Klein et al. [7]. In short, we normalized the expression matrix on a per individual per cell type basis. We then created pseudobulk expression matrices for each broad cell type (excitatory neurons, oligodendrocytes, inhibitory neurons, astrocytes, oligodendrocyte precursor cells, microglia, pericytes, and endothelial cells) by calculating the average expression per gene and per individual. We included only genes that showed expression in at least 90% of the cell types and then performed a center and scaling per sample.

### Cell type expression enrichment of PICs

The BLUEPRINT and ROSMAP expression in different blood and brain cell types was used to evaluate if the genes associated to PICs show cell type-specific expression patterns. The same top 200 associated genes were used for the gene set enrichment analysis. Per cell type, we calculated the mean expression of the associated genes to evaluate if the PICs reflect cell type composition differences.

### Replication of cell type context PIC ieQTLs in single-nucleus eQTLs

We replicated the ieQTLs for the first five PICs in brain single-nucleus derived eQTL summary statistics published by Bryois et al. [34]. We overlapped their summary statistics with the PIC ieQTLs and found that, dependent on the cell type, between 3796 and 5774 overlapped. We calculated a BH-FDR on the *p*-values of the ieQTLs that were significant in the respective cell type. Furthermore, we calculated three different measurements of agreement (AC, *π*_1_ [36], and *R*_*b*_ [35]) using the ieQTLs that had a significant interaction with the PIC. Since the summary statistics did not include standard errors or MAF values, we predicted beta and standard errors using the MetaBrain Cortex-EUR MAF together with the eQTL sample size, beta, and *p*-value [44] from Bryois et al. to calculate *R*_*b*_ metrics. To calculate the *π*_1_, we took the Bryois et al. eQTL *p*-values and calculated the proportion of true null *p*-values (*π*_0_) with the pi0est function from qvalue [45] and subsequently calculated *π*_1_ as 1 − *π*_0_.

### Replication of brain PIC ieQTLs across datasets

In order to test if PIC ieQTLs are robust across datasets, we replicated the ieQTLs identified in the brain (MetaBrain EUR; *n* = 2440) in the MetaBrain AFR subset (*n* = 311). We used the expression matrix as described for the gene set enrichment analysis and prepared the gene expression for AFR in an identical manner. We did not run PICALO on the AFR samples, but rather we first calculated the Pearson correlation between PIC values and gene expression levels in the EUR subset. In the AFR subset, we then used a dot product to associate the AFR gene expression levels with PIC-gene correlations observed in the EUR subset. This provides an estimate of the EUR PIC values in the AFR subset. We then performed ieQTL analysis in the EUR and AFR datasets as outlined in the “Detection of eQTL interacting with PICs or expression PCs” section, but in this case, we did not perform conditional analysis. Per PIC, we calculated a BH-FDR over the *p*-values of the ieQTLs that were significant in EUR. Furthermore, we calculated three different measurements of agreement (AC, *π*_1_ [36], and *R*_*b*_ [35]) as described in the replication with Bryois et al. Since both betas and standard errors were available for this replication, no estimation based on MAF and *p*-value was required.

### Supplementary Information

**Additional file 1: Figs. S1-S23.** Supplementary figures including legends.

**Additional file 2: Table S1.** Number of interaction eQTLs per expression PC or PIC in blood and brain.

**Additional file 3: Table S2.** Blood conditional PIC ieQTL summary statistics.

**Additional file 4: Table S3.** Brain conditional PIC ieQTL summary statistics.

**Additional file 5.** Supplementary note.

**Additional file 6: Table S4.** Blood annotation correlations.

**Additional file 7: Table S5.** Brain annotation correlations.

**Additional file 8: Table S6.** Blood and brain gene expression vs PIC correlations.

**Additional file 9: Table S7.** ToppGene gene list enrichment.

**Additional file 10: Table S8.** Replication of the MetaBrain EUR ieQTLs in AFR samples.

**Additional file 11.** Review history.

## Data Availability

The raw RNA-seq data of BIOS can be obtained from the European Genome-phenome Archive (EGA; accession EGAS00001001077 [52]). Genotype data are available from the respective biobanks: LLS (http://www.leidenlangleven.nl/; email: m.beekman@lumc.nl), LifeLines (https://lifelines.nl/lifelines-research/access-to-lifelines; email: llscience@umcg.nl), CODAM (email: m.vangreevenbroek@maastrichtuniversity.nl), and RS (https://www.erasmusmc.nl/en/research/core-facilities/ergo-the-rotterdam-study; email: m.a.ikram@erasmusmc.nl). The MetaBrain dataset comprised previously published human brain eQTL datasets. The majority of these datasets are available upon request or through online repositories after signing data access agreements. We have listed the mode of access for each of the included datasets below: TargetALS [53] TargetALS data was pushed directly from the New York Genome Center (https://www.targetals.org/) to our SFTP server. CMC [54] CMC data was downloaded from https://www.synapse.org/ using the Synapse Client (https://python-docs.synapse.org/), accession code: syn2759792 [55]. GTEx [56] GTEx was downloaded from Sequence Read Archive (SRA) using fastq-dump of the SRA toolkit (http://www.ncbi.nlm.nih.gov/Traces/sra/sra.cgi?cmd=show&f=software&m=software&s=software). Access has been requested and granted through dbGaP, accession code: phs000424.v7.p2 [57]. AMP-AD [58] AMP-AD data has been downloaded from Synapse, accession code: syn2580853 [59]. snRNA-seq was collected using Synapse, accession code: syn18485175 [60]. ENA [61] ENA data has been downloaded from the European Nucleotide Archive. The identifiers of the 76 included studies and 2021 brain samples are listed in de Klein et al. [7]. CMC_HBCC [54] CMC_HBCC data was downloaded from https://www.synapse.org/ using the Synapse Client (https://python-docs.synapse.org/), accession code: syn10623034 [62]. BrainSeq [63] BrainSeq data was downloaded from https://www.synapse.org/ using the Synapse Client (https://python-docs.synapse.org/), accession code: syn12299750 [64]. UCLA_ASD [65] UCLA_ASD data was downloaded from https://www.synapse.org/using the Synapse Client (https://python-docs.synapse.org/), accession code: syn4587609 [66]. BrainGVEx [65] BrainGVEx data was downloaded from https://www.synapse.org/ using the Synapse Client (https://python-docs.synapse.org/), accession code: syn4590909 [67]. BipSeq [65] BipSeq data was downloaded from https://www.synapse.org/ using the Synapse Client (https://python-docs.synapse.org/), accession code: syn5844980 [68]. NABEC [69] NABEC data was downloaded from dbgap, accession code: phs001301.v1.p1 [70]. BLUEPRINT [42] expression data was downloaded from http://blueprint-data.bsc.es/. The *PICALO* software is available at https://github.com/molgenis/PICALO[71] under the BSD 3-Clause “New” or “Revised” License. The code version used for the manuscript, as well as any custom code, is available at https://doi.org/10.5281/zenodo.8172196[72]. Software packages used in PICALO and throughout this manuscript include the following: Python (v3.7.4) R (v4.0.3), qvalue [45] (v2.15.0), numpy [73] (v1.19.5), pandas [74] (v1.2.1), scipy [75] (v1.6.0), statsmodels [76] (v0.12.2), matplotlib [77] (v3.3.4), seaborn [78] (v0.11.1), scikit-learn [79] (v0.24.1), and upsetplot [80, 81] (v0.4.1).
