# Supplementary material for: PICALO: principal interaction component analysis for the identification of discrete technical, cell-type, and environmental factors that mediate eQTLs
Source: Genome Biol. 2024 Jan 22;25:29. doi: 10.1186/s13059-023-03151-0 (PMC10802033; doi:10.1186/s13059-023-03151-0)
Supplement: Supplementary file 1 — Additional file 1: Figs. S1-S23. Supplementary figures including legends. [file 13059_2023_3151_MOESM1_ESM.pdf]

Fig. S1. Correlation between RNA-seq alignment metrics and cell type proportions

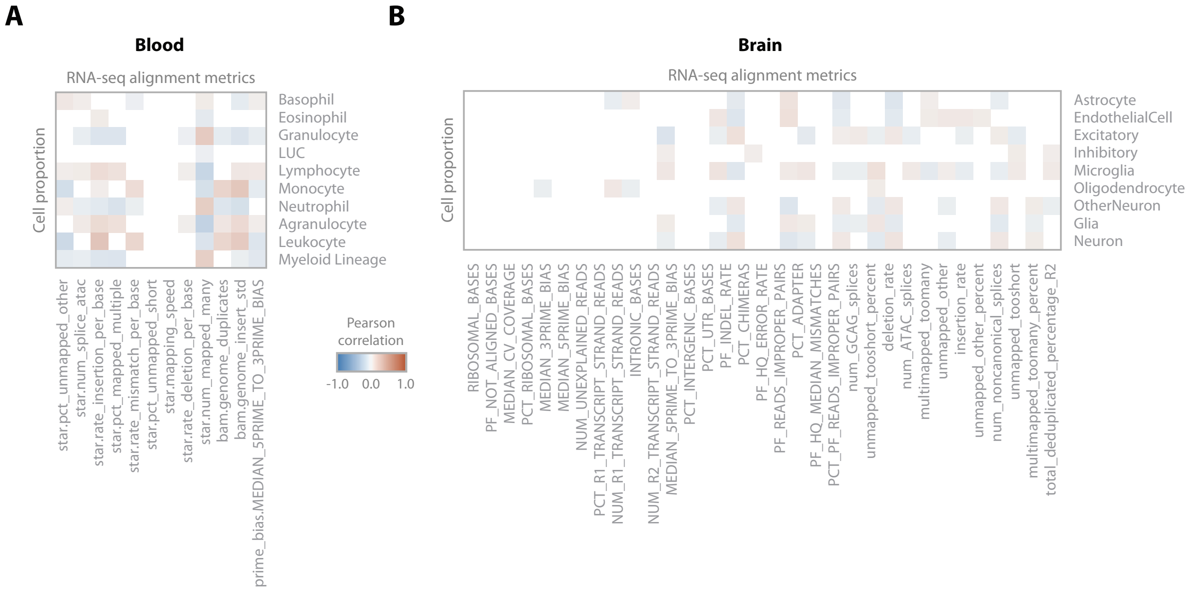

Pearson correlations between RNA-seq alignment metrics and measured cell type proportions in blood (A), and predicted cell type proportions in brain (B). The correlations in brain are less evident, most likely because RNA-seq alignment metrics were explicitly corrected for prior to predicting cell proportions in this dataset.

Fig. S2. Unique and overlapping PIC interaction eQTLs

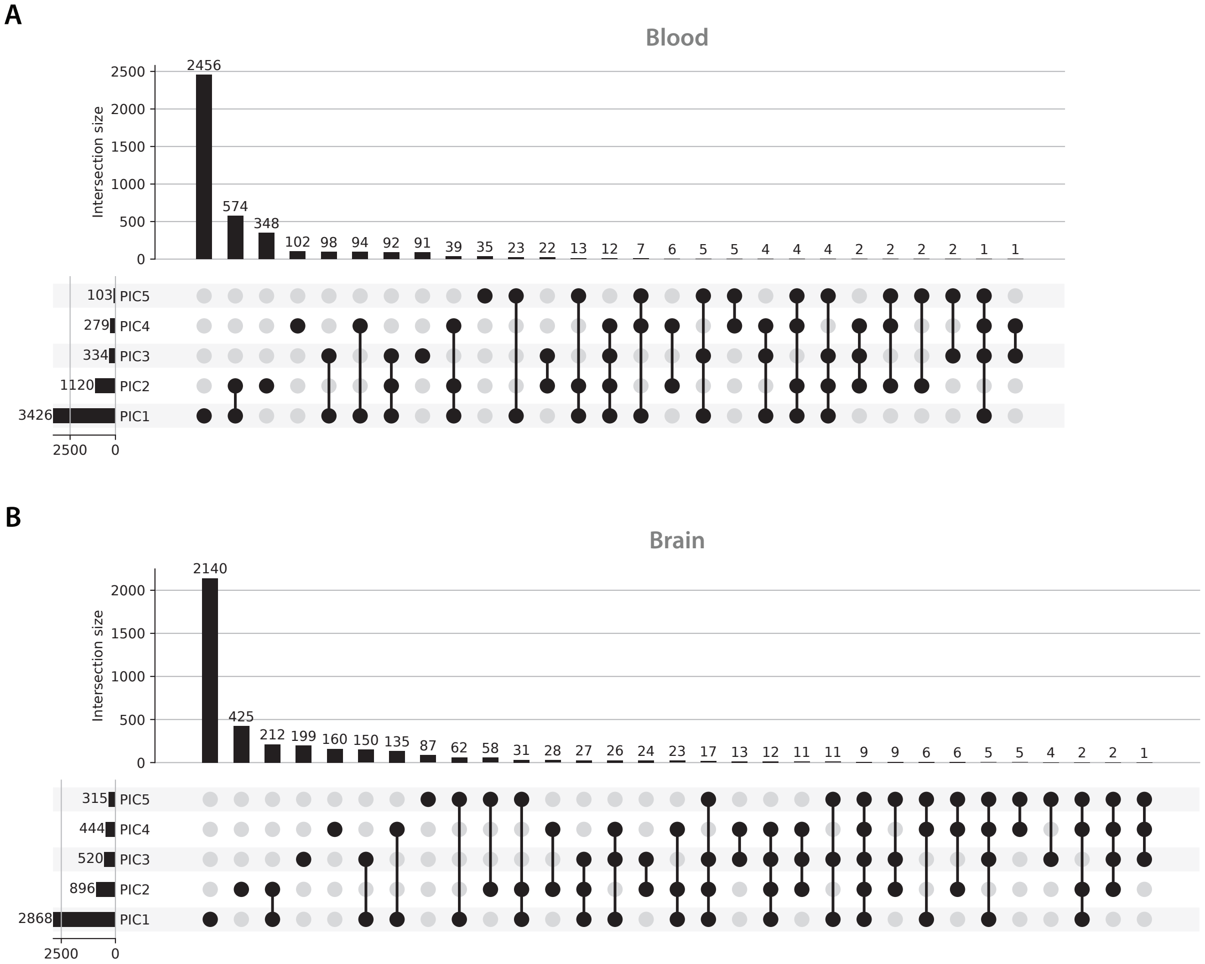

Unique and overlapping PIC ieQTLs for the first five PICs in blood (A) and brain (B). The majority of eQTLs interact with a single PIC.

Fig. S3. Correlation between PICs

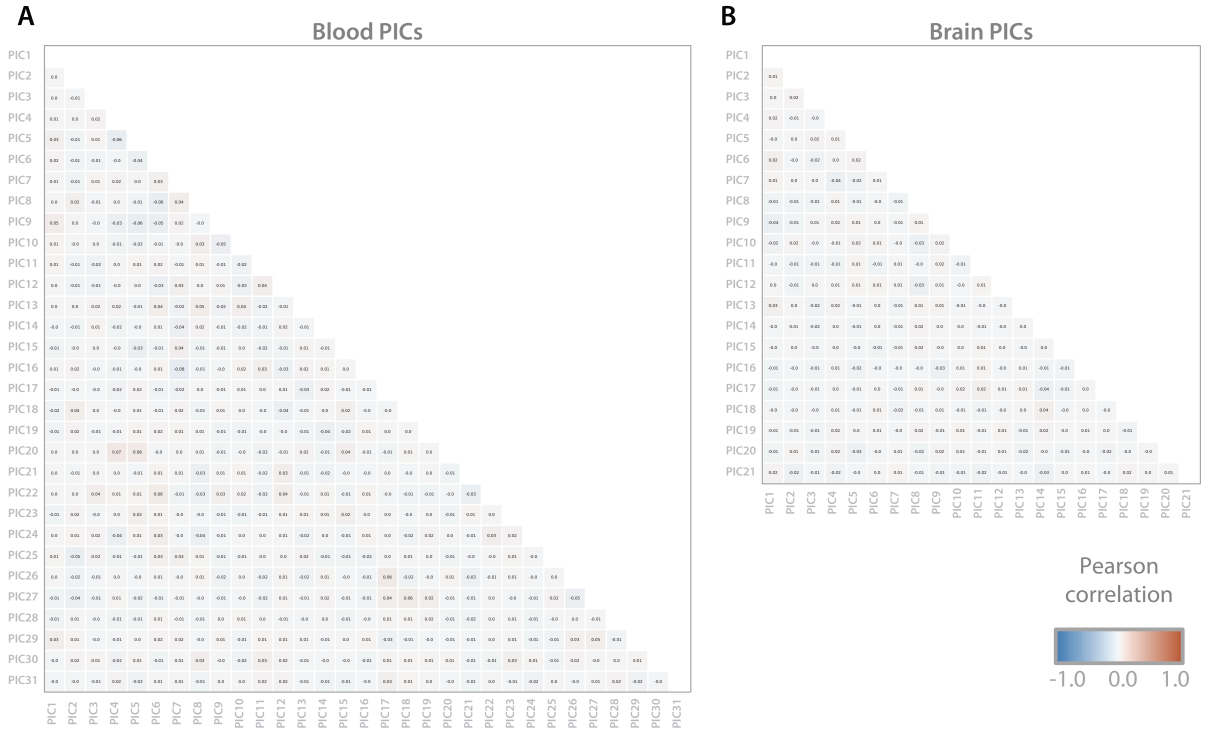

Pearson correlation between identified PICs in blood (A) and brain (B). None of the PICs are correlaed in either dataset (max. Pearson r <0.07).

Fig. S4. Correlation between optimized PIC and the starting position

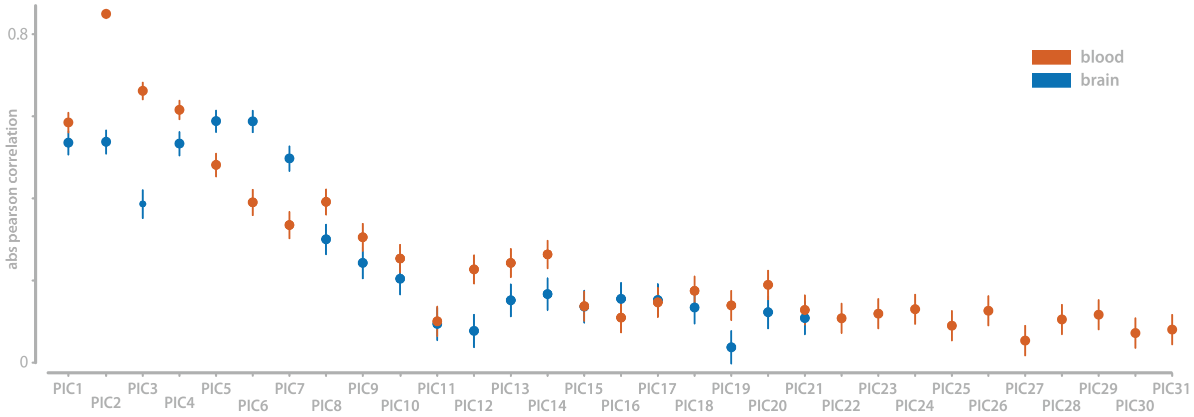

The Pearson correlation between the starting position (expression PC; before optimization) and the resulting PIC (after optimization) for blood and brain. The error bars indicate the 95% confidence interval. The correlation before and after optimization decreases as the PICs capture less interaction variance.

Fig. S5. Simulation analysis

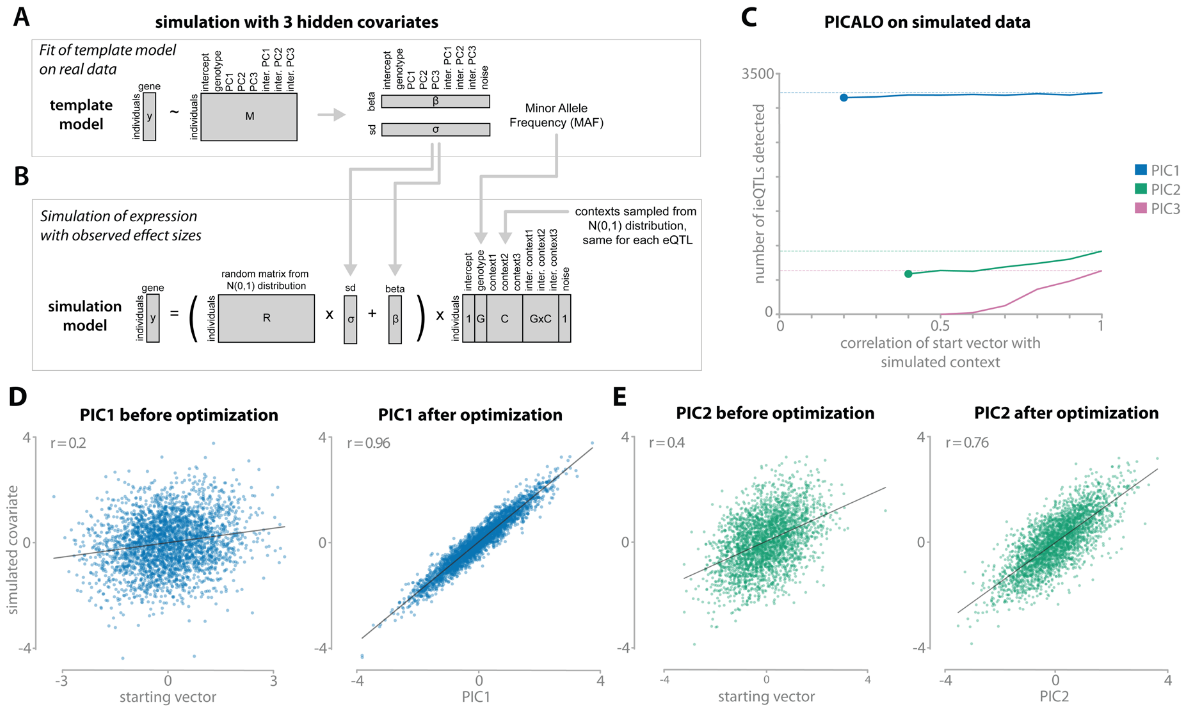

(A, B) Flowchart indicating discovery and replication scheme to simulate interaction eQTL data. (A) We generate a template model using real data from which the effect sizes, standard deviations, and minor allele frequency (MAF) are used to generate simulated data. (B) Genotypes are randomly generated using the observed minor allele frequency, three random covariates are sampled from a normal distribution and are consistent over all simulated eQTLs. The expression is then calculated by a weighted multiplication of the simulated model by the observed effect sizes and standard deviations. The dots indicate the example visualized in panel D (blue) and E (green). (D, E) Comparison between the start vector (left x-axis), identified PIC (right x-axis) and the simulated context (y-axis) for PIC1 using a starting vector of ±0.2 (D) and PIC2 using a starting vector of ±0.4 (E). The r value denotes the Pearson correlation.

Fig. S6. Detection of ieQTLs for different sample sizes and effect sizes

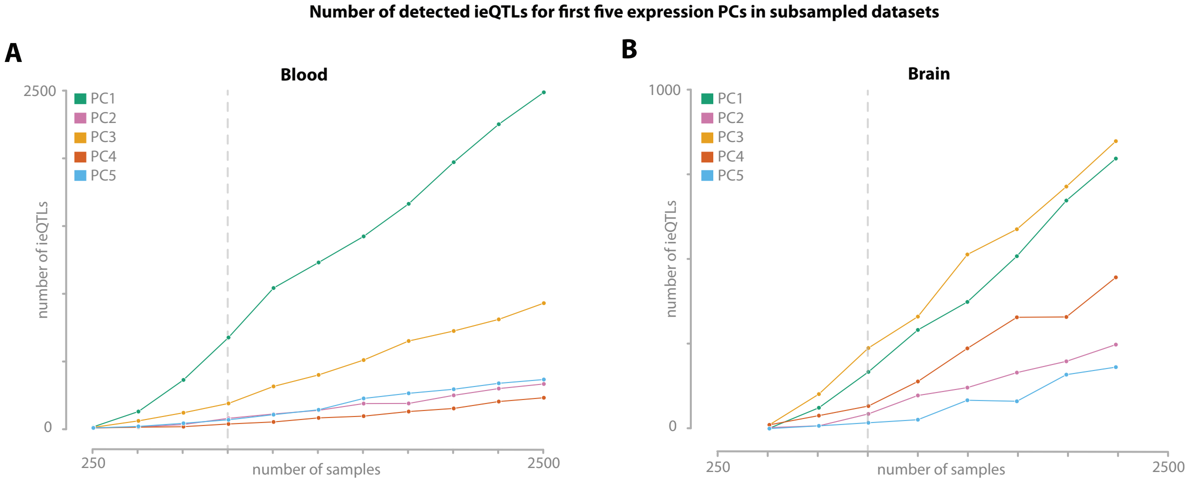

Comparison of the influence of dataset sample size on the number of detected ieQTLs using expression PCs as covariates in blood (A) and brain (B). There is a near linear relationship between the sample size, the effect size of the context, and the number of significant ieQTLs. These results indicate, given these effect sizes, that a sufficient number of significant ieQTLs are found for PICALO to yield reliable PICs when the number of samples is approximately 1,000 or more.

Fig. S7. Correlation between identified PICs using different starting positions

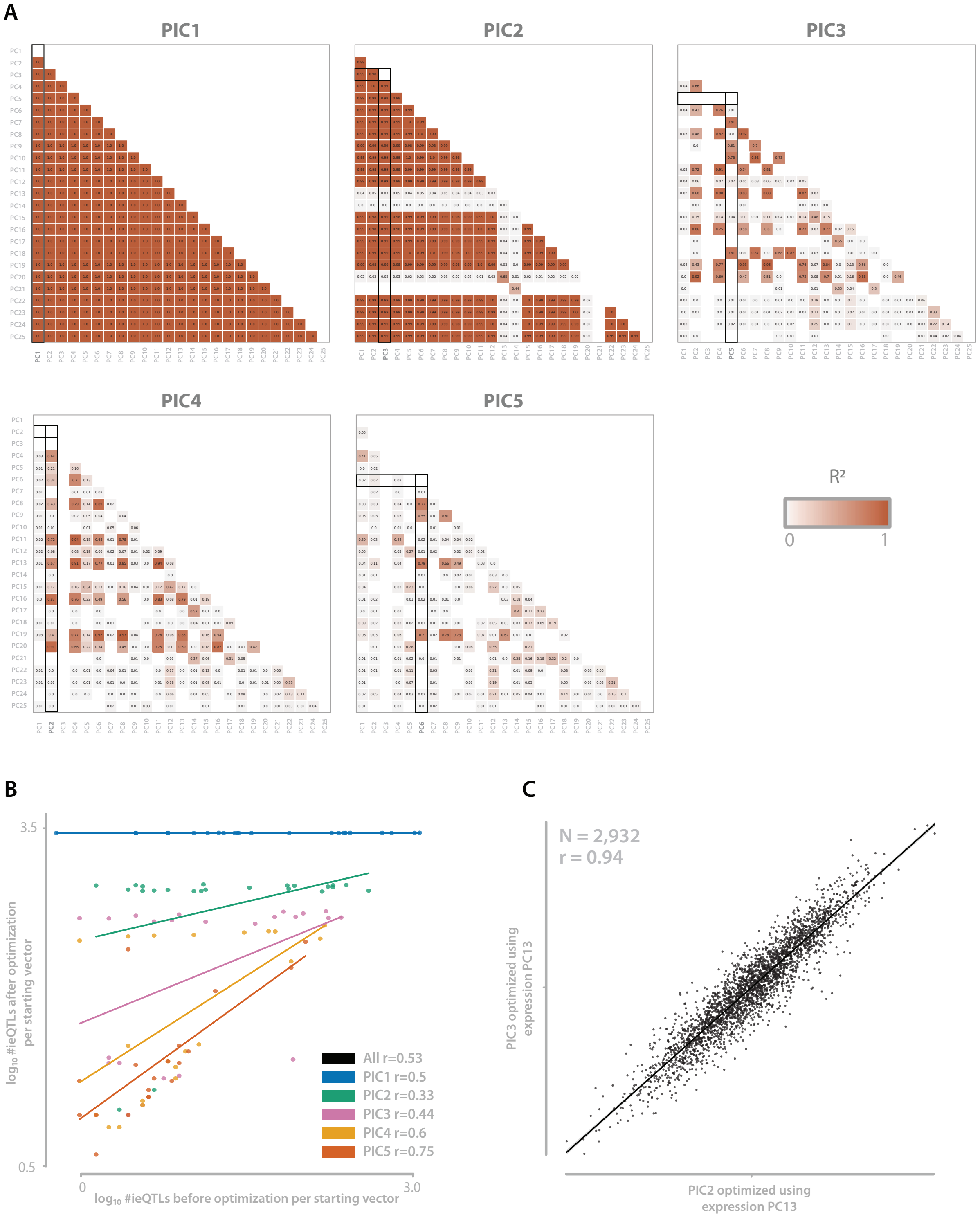

(A) Comparison of PICs using different expression PCs as starting position for maximization. The first five PICs are tested, for each PIC the starting position (i.e. expression PC) with the highest number of ieQTLs before optimization is used as PIC and is corrected for before analyzing PIC2 (starting positions used are PIC1=PC1, PIC2=PC3, PIC3=PC5, PIC4=PC2, PIC5=PC6). For each of starting positions the resulting outcome is compared to all the other outcomes when using different starting positions. Each cell contains the R^2 of these comparisons. Only correlations with a BH-FDR<0.05 are shown. PIC1 for example, shows that regardless of which starting position is used for optimization, the resulting outcome is the same. (B) Visualization of the relationship between the number of identified ieQTLS before (x-axis) and after (y-axis) optimization. For the top PICs there is no relation between the number of ieQTLs before and after optimization. However, this dependence increases as the proportion of interaction variance the PICs capture decreases. (C) Comparison of PIC2 optimized using expression PC13 as starting position versus PIC3 optimized using expression PC13. The r value denotes the Pearson correlation. Note that the PICALO outcome for PIC2 using expression PC13 was not regressed out when accessing PIC3 since a different PC had more ieQTLs prior to optimization. The PIC2-PC13 outcome, which is likely a local minimum, can therefore be reidentified when optimizing expression PC13 for PIC3, highlighting the robustness of the method.

Fig. S8. ieQTL replication within dataset

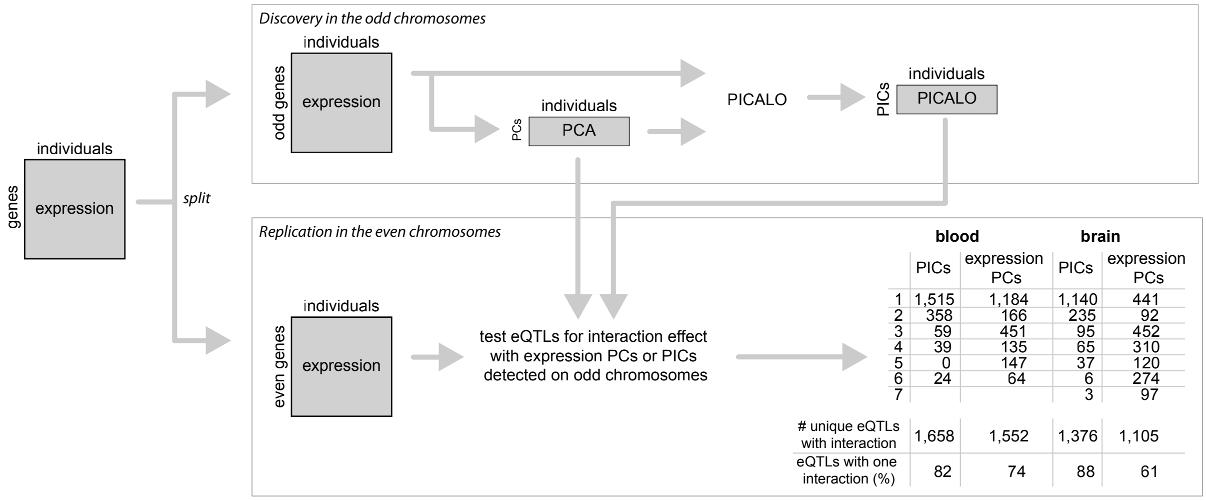

	blood		brain	
	PICs	expression PCs	PICs	expression PCs
1	1,515	1,184	1,140	441
2	358	166	235	92
3	59	451	95	452
4	39	135	65	310
5	0	147	37	120
6	24	64	6	274
7			3	97
# unique eQTLs with interaction	1,658	1,552	1,376	1,105
eQTLs with one interaction (%)	82	74	88	61

Graphic overview of the replication analysis within each dataset. The expression data is split in even and odd chromosomes. We continue pre-processing as normal using only the genes on the odd chromosomes, first applying PCA for an initial guess of eQTL context and subsequently applying PICALO to identify PICs. We then use the PCs and PICs separately to map ieQTLs with the eQTLs on the even chromosomes.

Fig. S9. Brain PIC and expression PC correlations with estimated RNA-seq sample

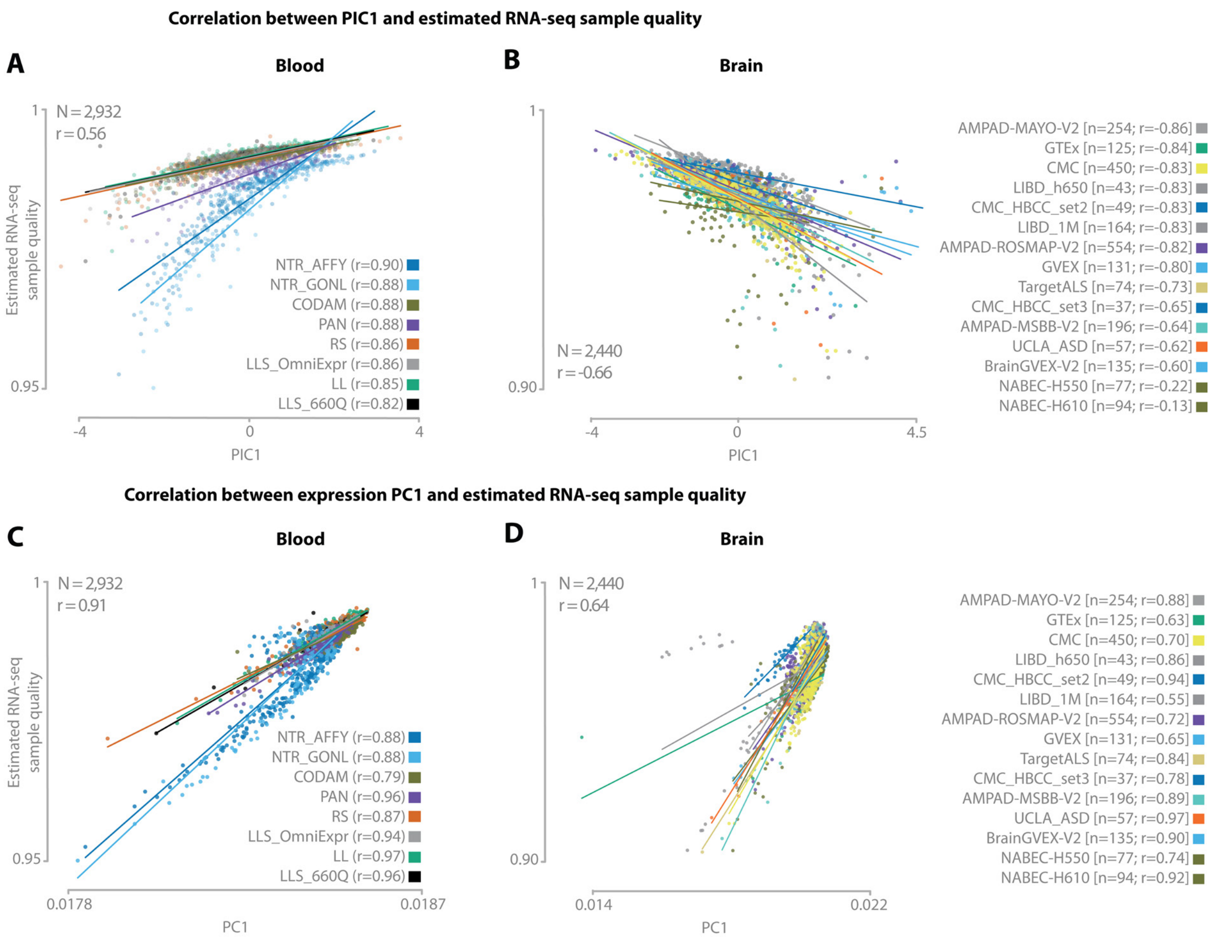

Regression plot showing the correlation between PIC1 (A and B) and expression PC1 (C and D) and estimated RNA-seq sample quality calculated as the per sample expression correlation with the overall average expression in blood and brain.

Fig. S10. Brain PIC and expression PC correlations with RNA-seq alignment metrics

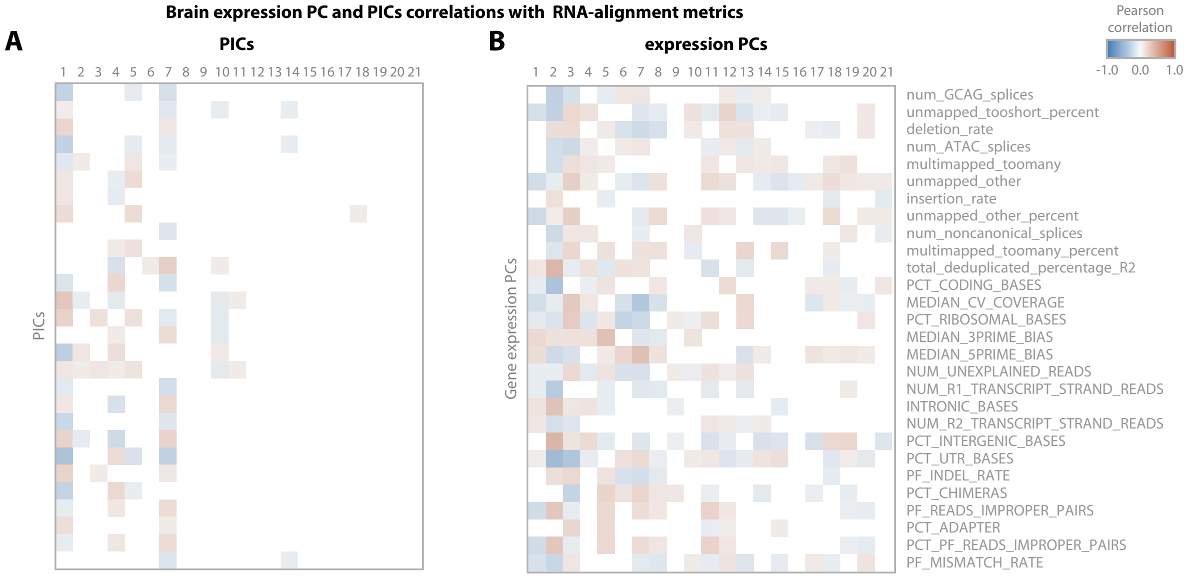

Pearson correlation heatmaps correlating PICs (A) as well as expression PC (B) to RNA-seq alignment metrics in brain. The correlation p-values are corrected for multiple testing with Benjamini-Hochberg and only correlations with a FDR <0.05 are shown. Note that many of the expression PCs correlate significantly with RNA-seq alignment metrics while only a limited number of PICs show significant correlations.

Fig. S11. Cell type proportion in blood (measured) and brain (predicted)

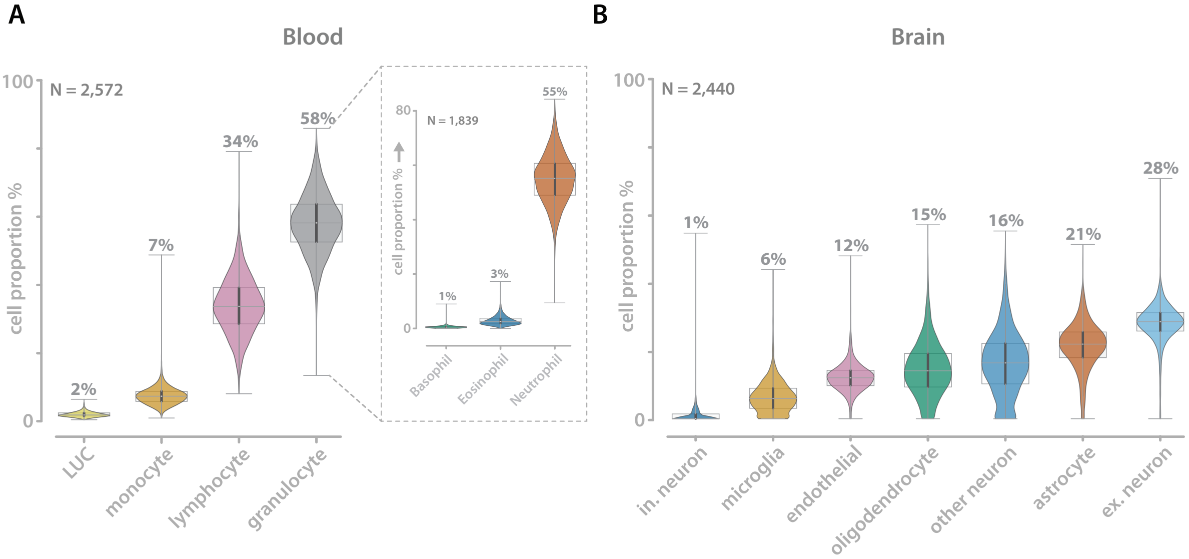

(A) Measured cell type proportions for the blood samples. For a subset of samples (n=1,839) the granulocytes are further distinguished into sub cell types. (B) Predicted cell type proportions for the brain samples as described by de Klein et al..

Fig. S12. Gene set enrichment in BLUEPRINT data

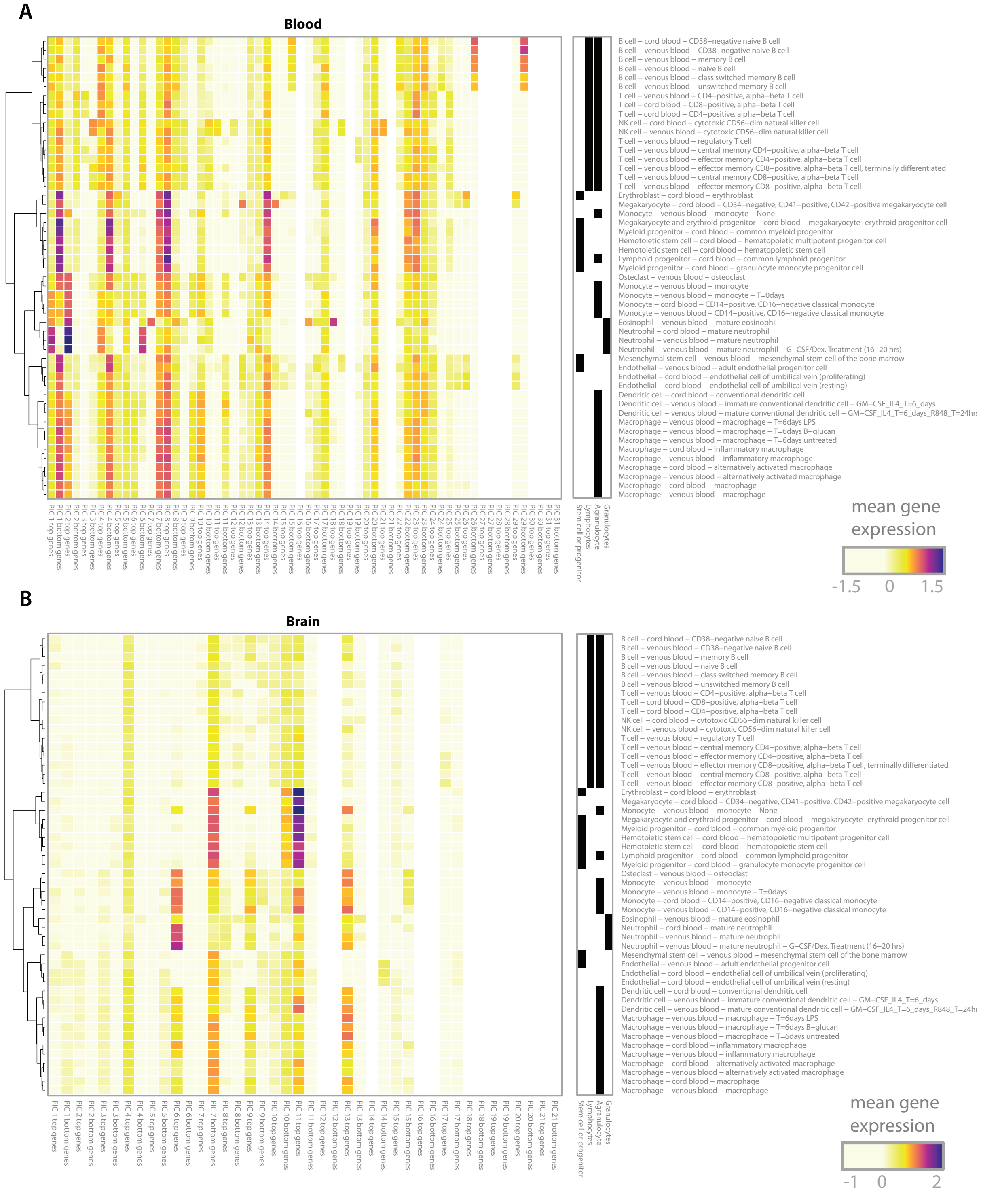

Mean gene expression of purified BLUEPRINT RNA-seq expression for the top 200 positively and top 200 negatively correlated genes in blood (A) and brain (B). The FPKM+1 BLUEPRINT expression data is log_2 transformed and center and scaled per sample.

Fig. S13. Brain gene set enrichment in ROSMAP single-nucleus data

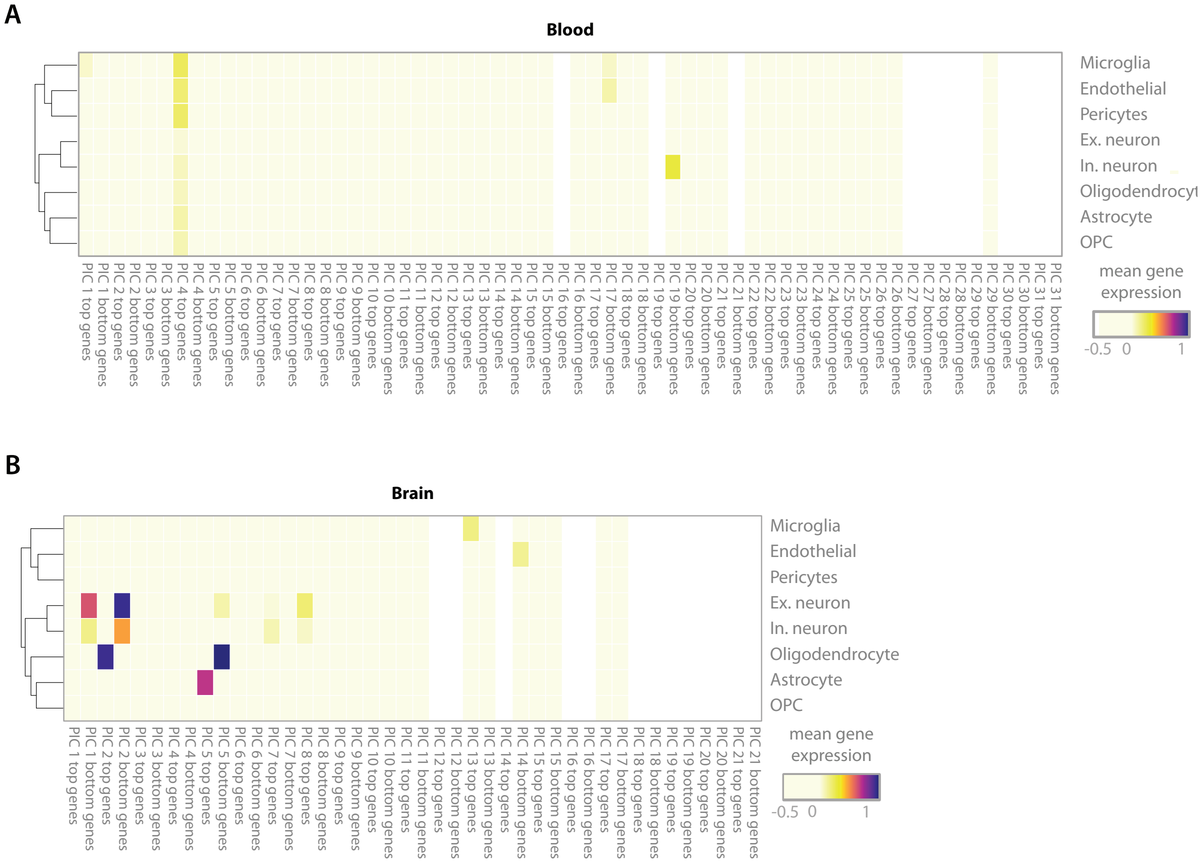

Mean gene expression of the ROSMAP single-nucleus RNA-seq expression for top 200 positively and top 200 negatively correlated genes in blood (A) and brain (B). The ROSMAP expression data is log_2 transformed and center and scaled per sample.

Fig. S14. PIC3 correlated with antibody PC describing prior CMV infection

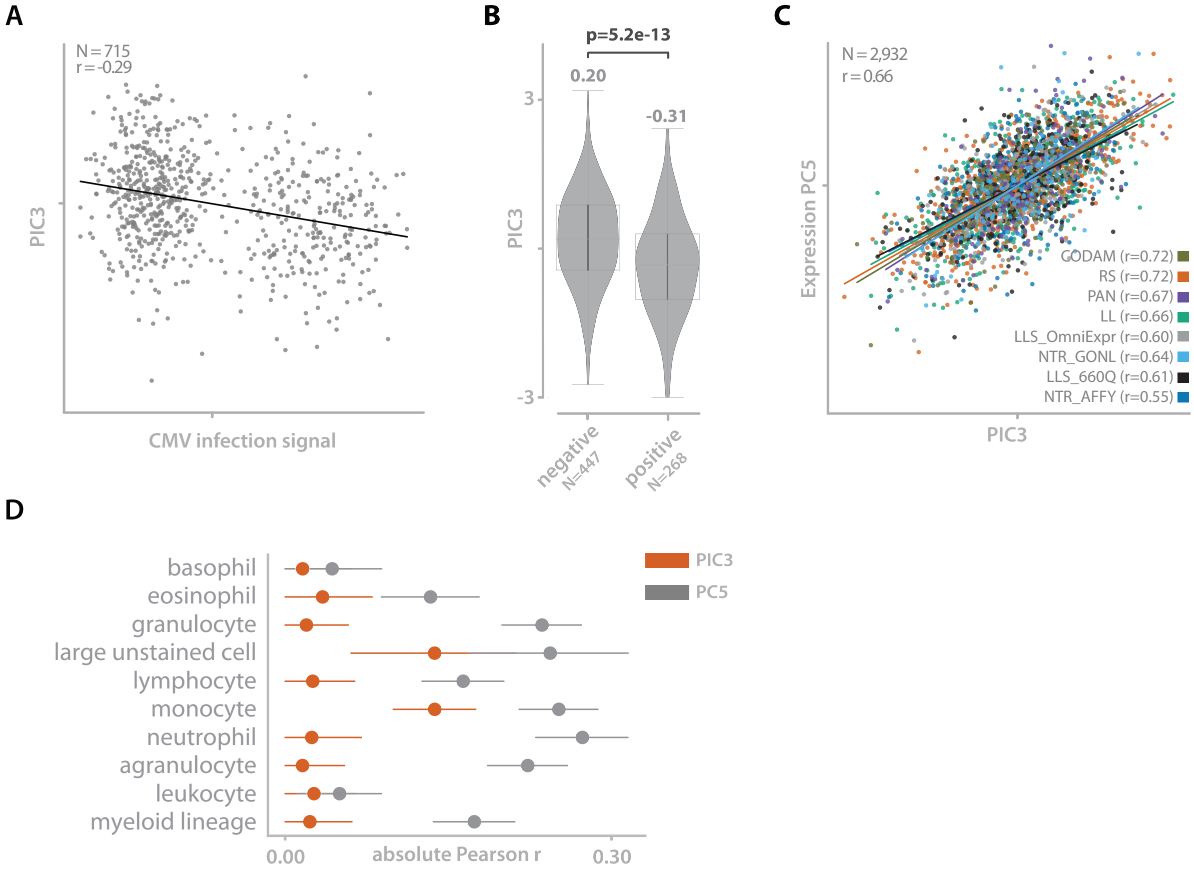

(A) Regression plot comparing the PIC3 to CMV infection signal (aggregate of multiple IgG antibody profiles) for the LifeLines (LL) cohort. (B) Violin plot comparing the PIC3 scores for samples with a negative or a positive CMV infection signal. The significance between the two groups is calculated using a two-sided Mann–Whitney U test. (C) Comparison of PIC3 and expression PC5, both correlating with CMV infection signal. (D) Forest plot showing the absolute Pearson correlations and 95% confidence intervals for PIC3 and expression PC5 compared to cell type proportions. PIC3 shows very low correlations while expression PC5 shows higher correlation, suggesting that PIC3 interactions are less biased by cell type confounding.

Fig. S15. Brain PIC ieQTL replication summary stats of replication in single-nucleus

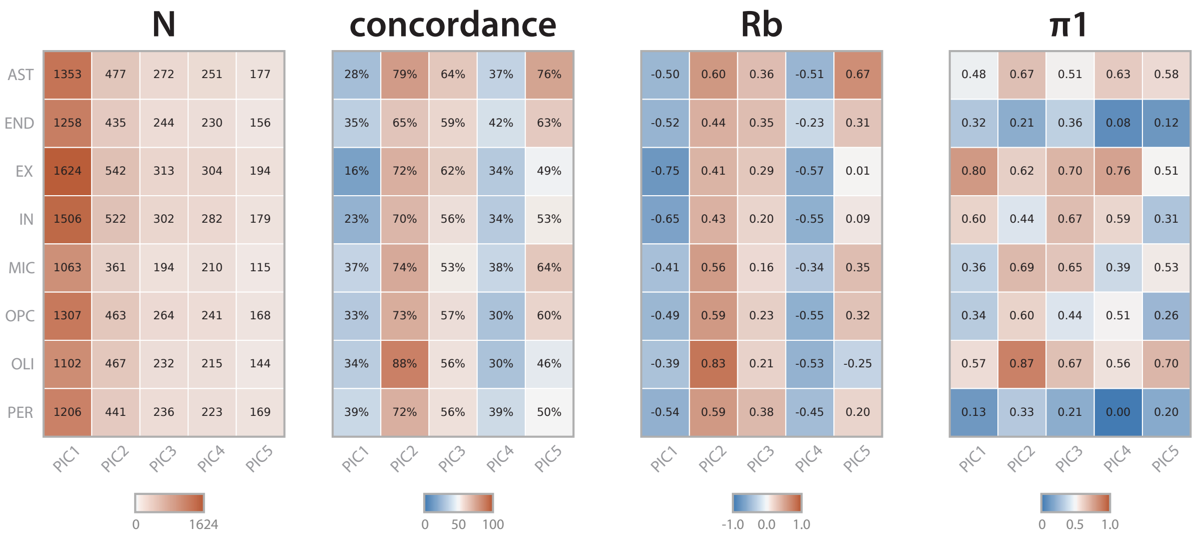

N	PIC1	PIC2	PIC3	PIC4	PIC5
AST	1353	477	272	251	177
END	1258	435	244	230	156
EX	1624	542	313	304	194
IN	1506	522	302	282	179
MIC	1063	361	194	210	115
OPC	1307	463	264	241	168
OLI	1102	467	232	215	144
PER	1206	441	236	223	169

concordance	PIC1	PIC2	PIC3	PIC4	PIC5
AST	28%	79%	64%	37%	76%
END	35%	65%	59%	42%	63%
EX	16%	72%	62%	34%	49%
IN	23%	70%	56%	34%	53%
MIC	37%	74%	53%	38%	64%
OPC	33%	73%	57%	30%	60%
OLI	34%	88%	56%	30%	46%
PER	39%	72%	56%	39%	50%

Rb	PIC1	PIC2	PIC3	PIC4	PIC5
AST	-0.50	0.60	0.36	-0.51	0.67
END	-0.52	0.44	0.35	-0.23	0.31
EX	-0.75	0.41	0.29	-0.57	0.01
IN	-0.65	0.43	0.20	-0.55	0.09
MIC	-0.41	0.56	0.16	-0.34	0.35
OPC	-0.49	0.59	0.23	-0.55	0.32
OLI	-0.39	0.83	0.21	-0.53	-0.25
PER	-0.54	0.59	0.38	-0.45	0.20

π1	PIC1	PIC2	PIC3	PIC4	PIC5
AST	0.48	0.67	0.51	0.63	0.58
END	0.32	0.21	0.36	0.08	0.12
EX	0.80	0.62	0.70	0.76	0.51
IN	0.60	0.44	0.67	0.59	0.31
MIC	0.36	0.69	0.65	0.39	0.53
OPC	0.34	0.60	0.44	0.51	0.26
OLI	0.57	0.87	0.67	0.56	0.70
PER	0.13	0.33	0.21	0.00	0.20

Replication of the first five MetaBrain PIC ieQTLs in the single-nucleus eQTL dataset by Bryois et al.. The first panel shows the number of overlapping eQTLs and each subsequent panel shows a different replication statistic. Cell types are abbreviated as follows: AST = astrocytes, END = endothelial cells, EX = excitatory neurons, IN = inhibitory neurons, MIC = microglia, OPC = oligodendrocyte precursor cell and committed oligodendrocyte precursor, OLI = oligodendrocytes, PER = pericytes. PIC2 ieQTLs replicate well in oligodendrocytes sn-eQTLs, while PIC5 replicates well in astrocytes.

Fig. S16. Brain PIC ieQTL replication in single-nucleus brain eQTLs

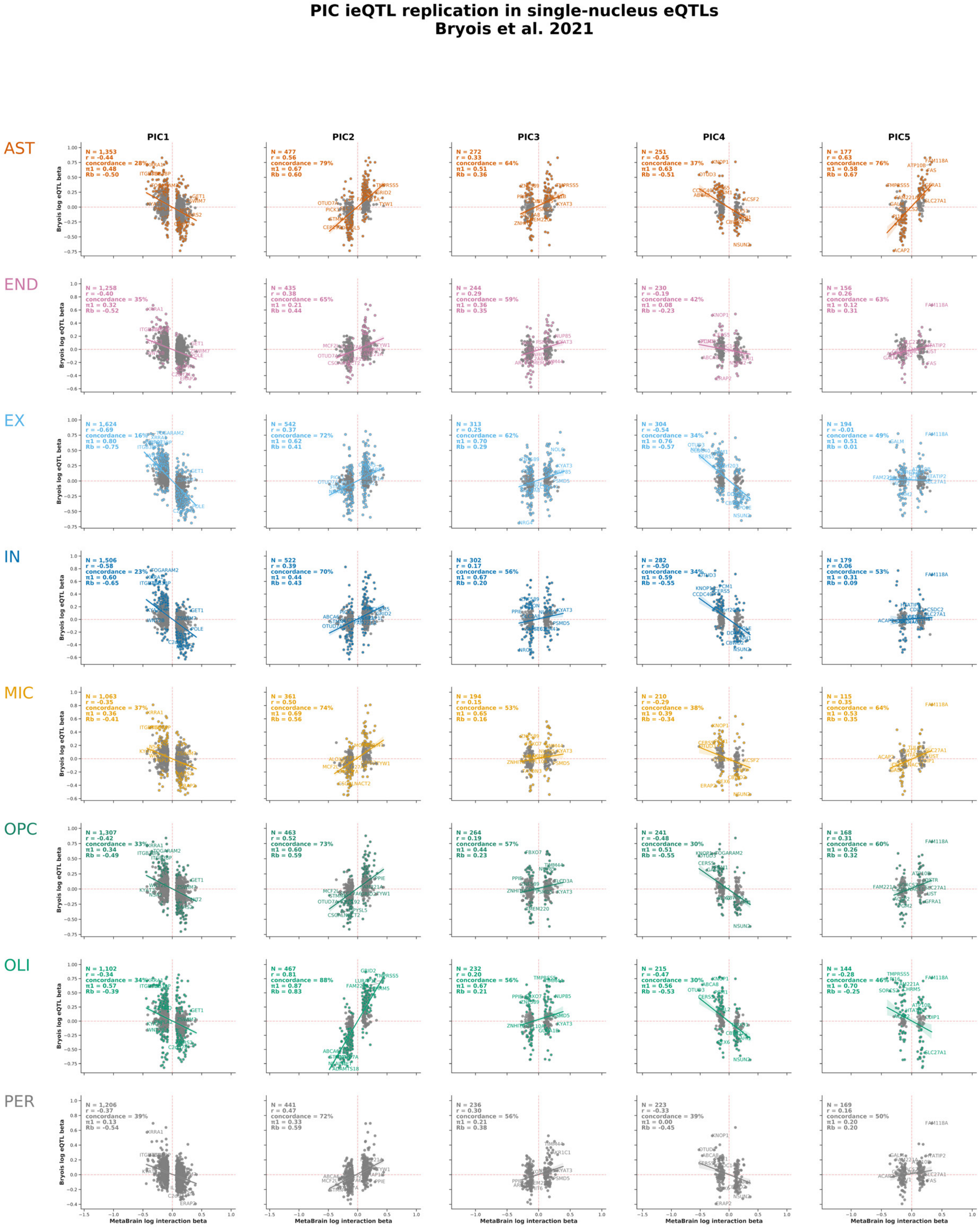

Replication of the first five MetaBrain PIC ieQTLs in the single-nucleus eQTL dataset by Bryois et al.. Each column denotes a different PIC, each row compares the PIC ieQTLs to the eQTLs identified in a distinct cell type. Cell types are abbreviated as follows: AST = astrocytes, END = endothelial cells, EX = excitatory neurons, IN = inhibitory neurons, MIC = microglia, OPC = oligodendrocyte precursor cell and committed oligodendrocyte precursor, OLi = oligodendrocytes, PER = pericytes. The shaded area denotes the 95% confidence interval. Each point denotes an eQTL of which the x-axis denotes the log interaction beta with a certain PIC, the y-axis denotes the log eQTL beta, and the color denotes if the eQTL effect significantly replicates (BH-FDR <0.05). Only significant interaction eQTLs are shown. The legend shows the sample size, Pearson correlation coefficient, the allelic concordance, the R_b *and finally the* π_1 *statistics.*

Fig. S17. Brain PIC ieQTL replication in cell-type interaction eQTLs

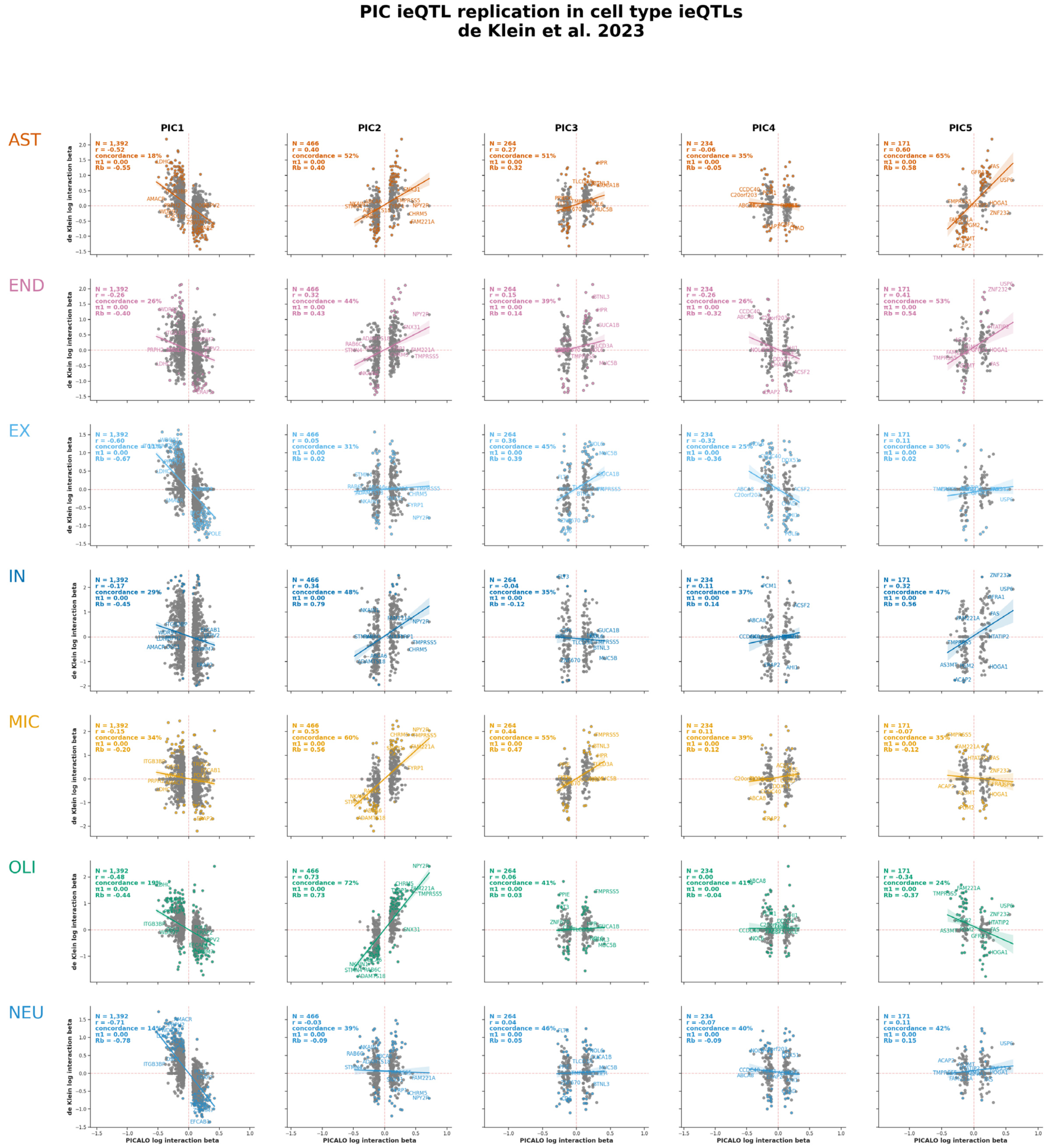

Replication of MetaBrain Cortex-EUR PIC ieQTLs in the MetaBrain cell type ieQTLs by Klein et al. Each column denotes a different PIC, each row compares the PIC ieQTLs to the eQTLs identified in a distinct cell type. Cell types are abbreviated as follows: AST = astrocytes, END = endothelial cells, EX = excitatory neurons, IN = inhibitory neurons, MIC = microglia, OPC = oligodendrocytes, NEU = other neurons. The shaded area denotes the 95% confidence interval. Each point denotes an eQTL of which the x-axis denotes the log interaction beta with a certain PIC, the y-axis denotes the log eQTL beta, and the color denotes if the eQTL effect significantly replicates (BH-FDR <0.05). Only significant interaction eQTLs are shown. The legend shows the sample size, Pearson correlation coefficient, the allelic concordance, the R_b and finally the π_1 statistics.

Fig. S18. Brain Cortex-EUR PIC replication in Cortex-AFR dataset

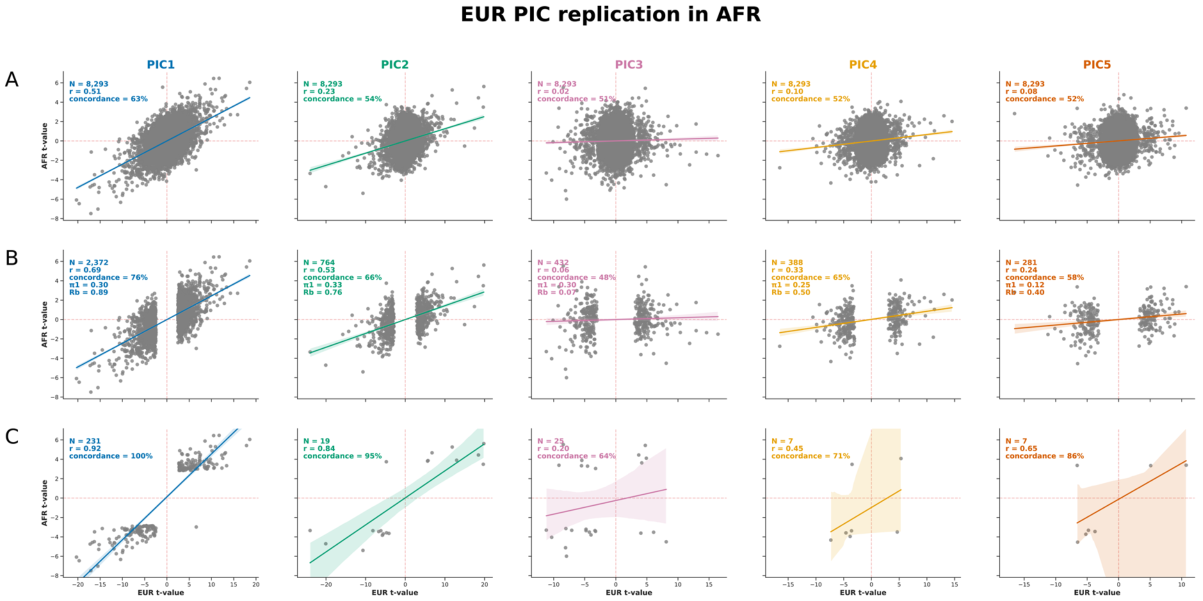

Replication of MetaBrain Cortex-EUR PIC ieQTLs in the MetaBrain Cortex-AFR dataset. Each figure in this plot represents a comparison between MetaBrain EUR (x-axis) and MetaBrain AFR (y-axis). Each dot represents one ieQTL, and the legend shows the sample size, Pearson correlation coefficient, the allelic concordance, and, if applicable, the R_b and π_1 statistics. Each column is a comparison between equivalent cell types in both datasets. Each row illustrates a different filtering on which eQTLs are shown. The x-axis always denotes the interaction t-value in EUR, the y-axis always denotes the interaction t-value in AFR. The shaded area indicates the 95% confidence interval. (A) All overlapping ieQTLs (B) ieQTLs filtered on being significant in EUR (C) ieQTLs filtered on being significant in both datasets.

Fig. S19. PICALO interaction eQTL identification and optimization

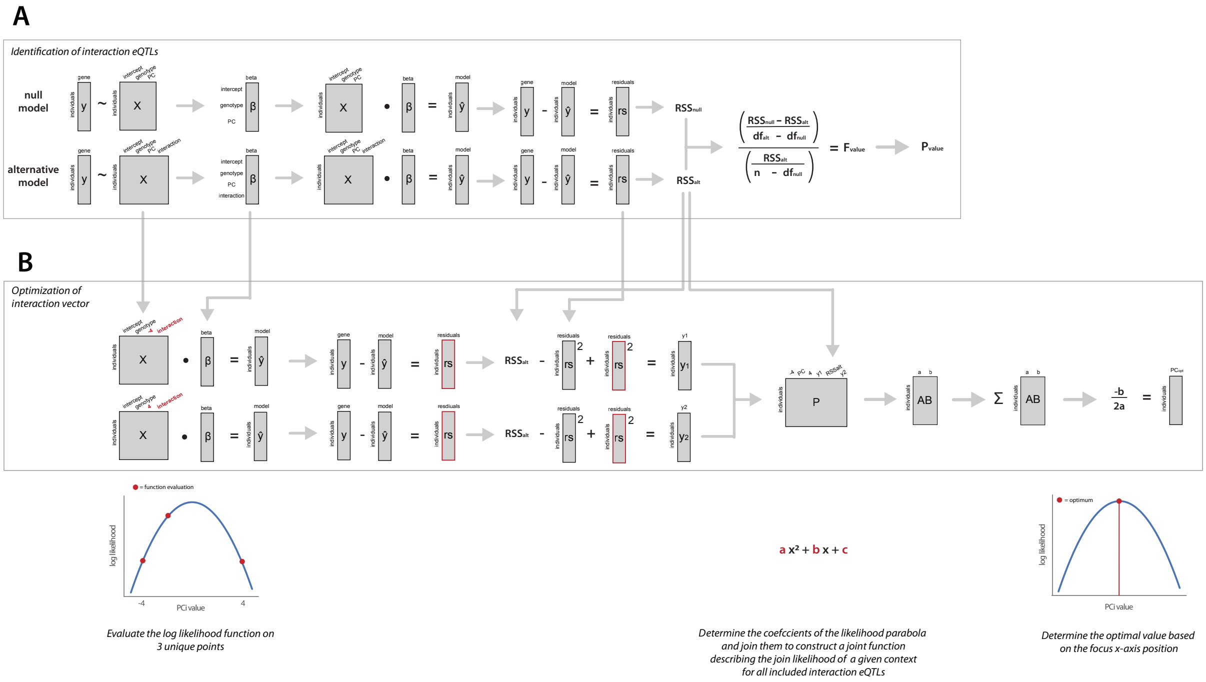

Interaction eQTL identification and optimization steps as implemented in PICALO. (A) the gene expression is modelled using two models; one without an interaction term (null model) and one with an interaction term (alternative model). By comparing the residual sum of squares of both models the significance of the interaction term can be determined using a F-test. (B) For the eQTLs that have a significant interaction, the optimal context value is determined per sample. For this the residuals are minimized over all included ieQTL per sample. Per sample and per ieQTL the alternative model is re-evaluated using -4 and +4 as context value. This, together with the starting position, gives three coordinates used to determine the unique second-degree polynomial that intersects these coordinates. The coefficients of the parabola are then summed together per sample over all ieQTLs to construct a combined function describing the joint log-likelihood. The optimal context value is then determined by calculating the focus of the combined function.

Fig. S20. Single interaction eQTL optimization example

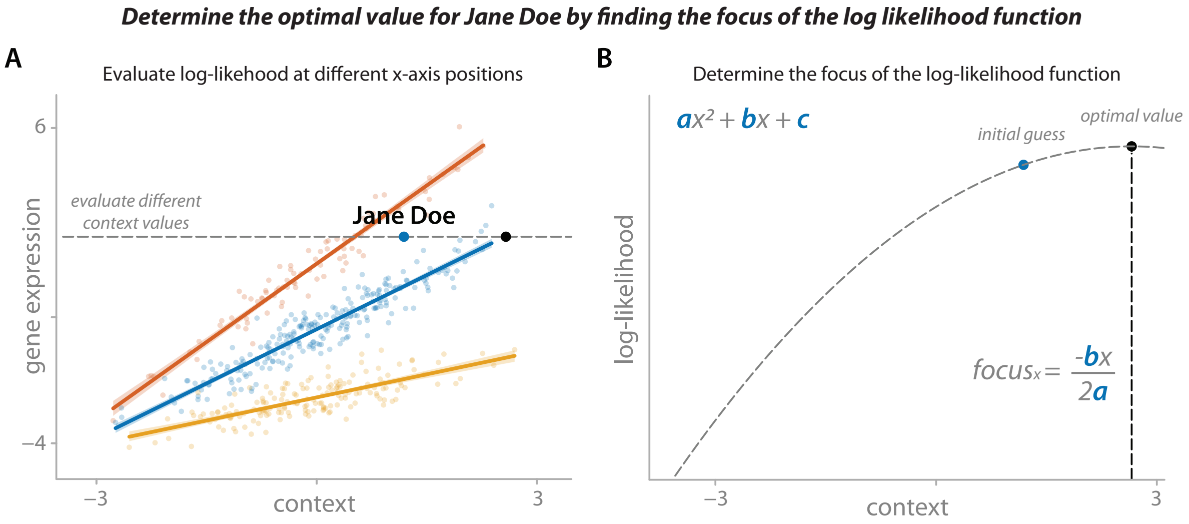

Cartoon example showing how the optimal context value for a single sample (Jane Doe) is determined when applying PICALO to a single ieQTL. (A) The ieQTL model is fitted over all samples. Per sample, the context value is adjusted (moved along the x-axis) and the change in log-likelihood is evaluated. Note that all other parameters, including the interaction beta, are not updated during this process. (B). The change in log-likelihood follows a second-degree polynomial function, the focus of which gives the context value with the maximum log-likelihood. To illustrate: in the case of a single ieQTL, this translates to sliding each sample in turn along the x-axis until it intersects with the regression line of its genotype group. In the case of multiple ieQTLs being optimized simultaneously (as is the case in PICALO), the log-likelihood functions are summed together, and the optimal context value is determined over the joint log-likelihood.

Fig. S21. Blood genotype MDS outlier removal

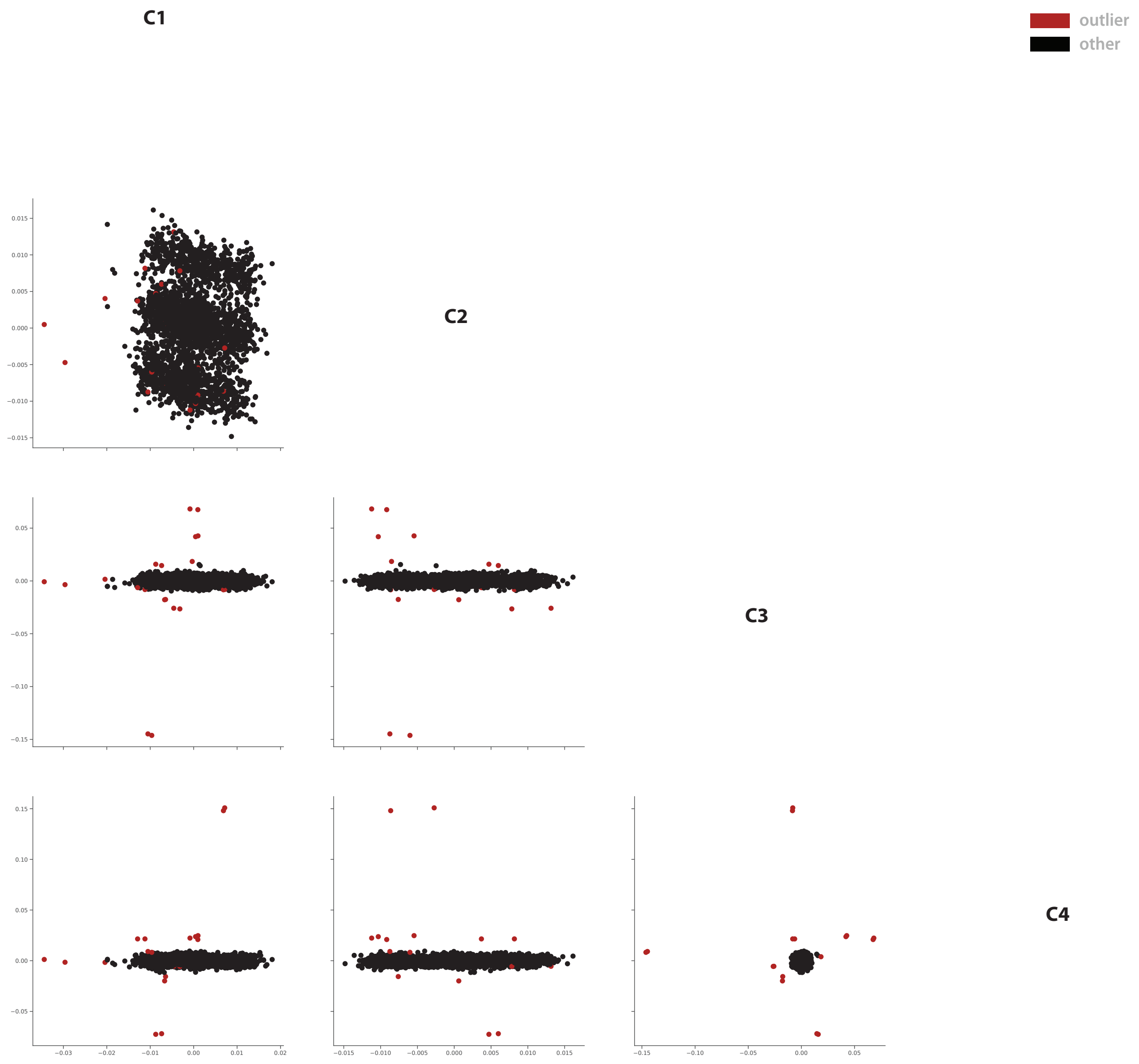

Exclusion of genotype outlier samples in blood. Samples that have absolute z-score >3 for any of the first four genotype MDS components are excluded (colored in red).

Fig. S22. Brain genotype MDS outlier removal

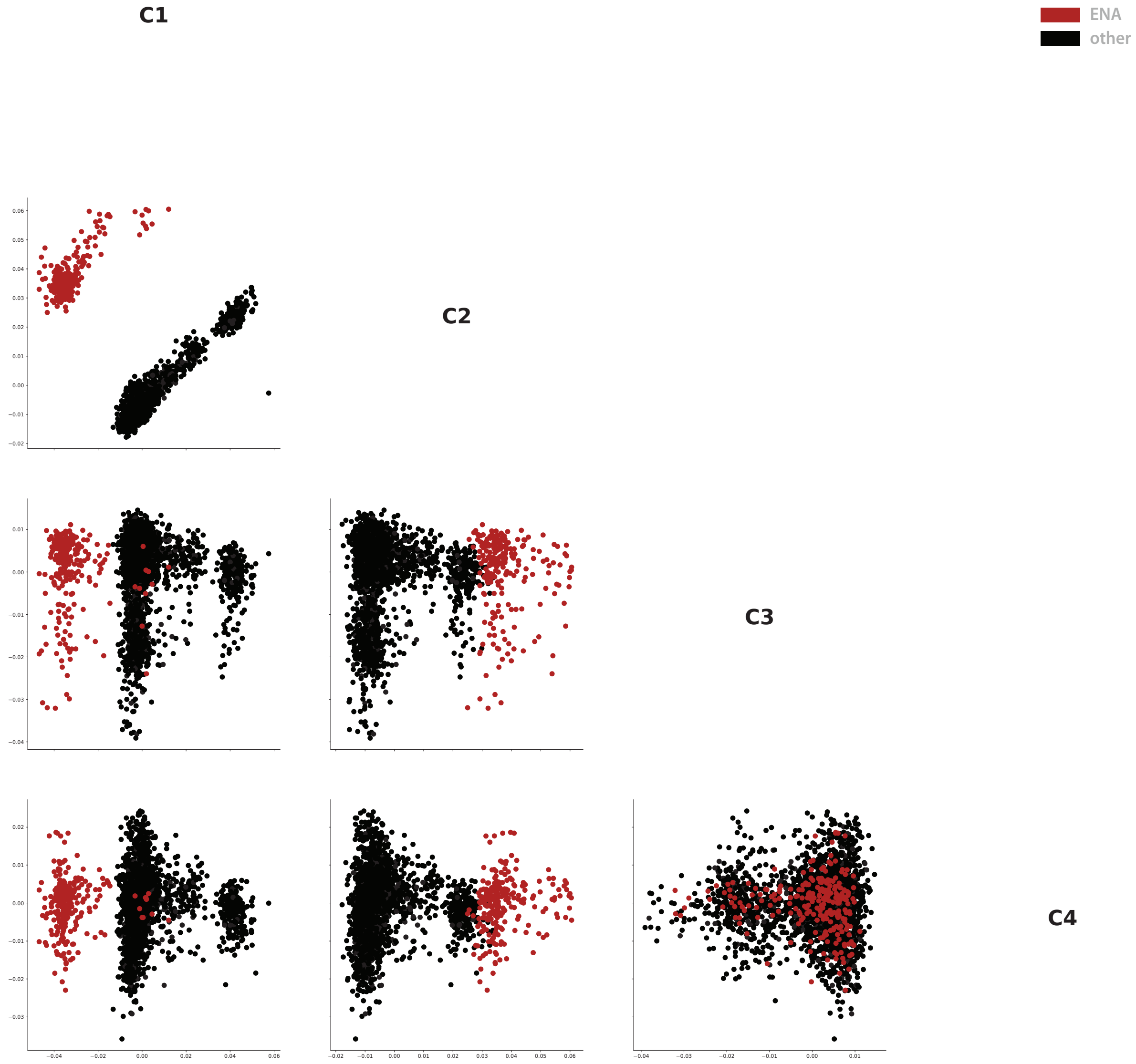

Exclusion of genotype outlier samples in brain. The ENA samples show to be a clear outlier (colored in red), most likely because these genotypes were derived from sequencing data.

Fig. S23. Genotype MDS and gene expression PCA for brain and blood

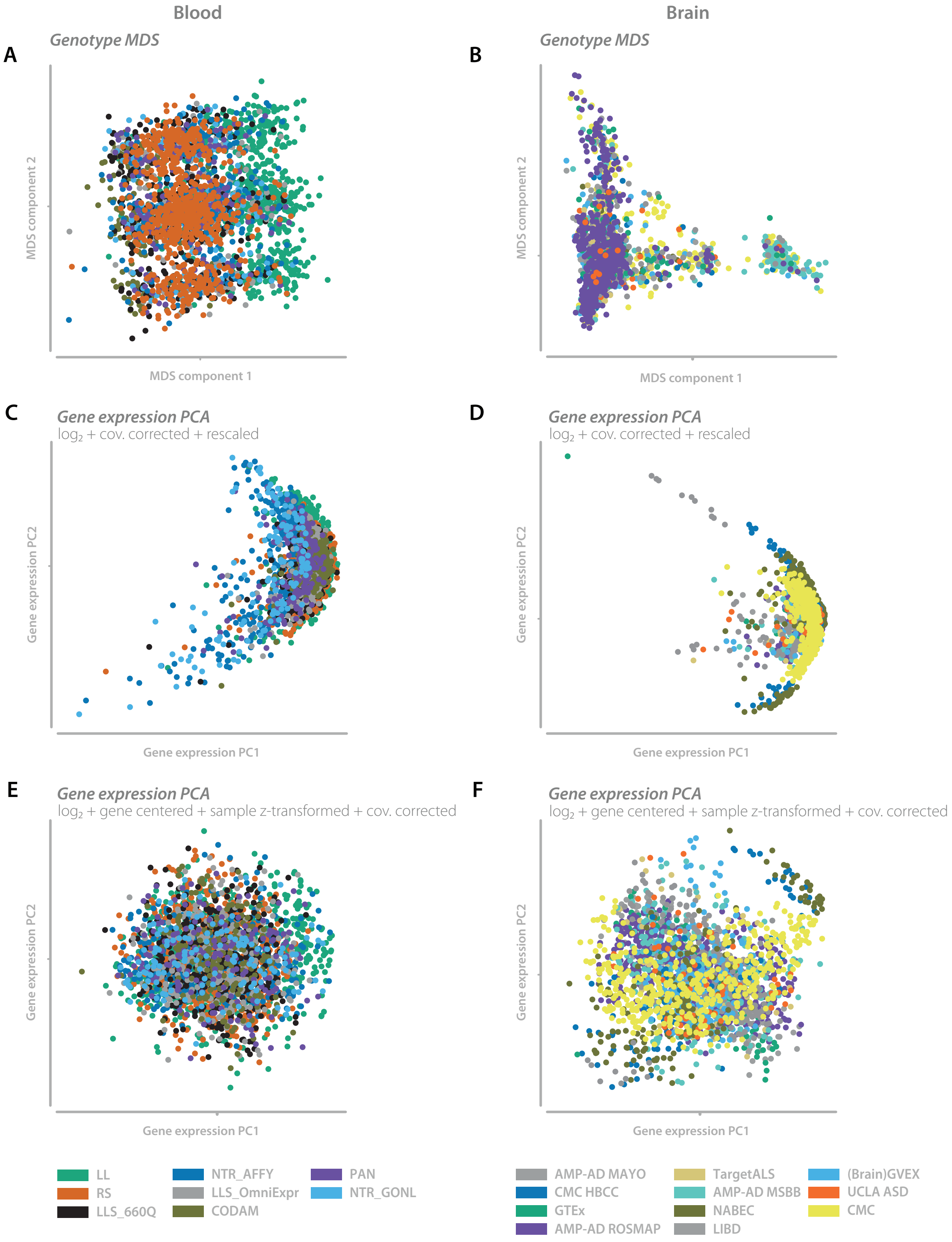

Genotype and gene expression dimensional reduction plots for blood and brain after removal of outliers. (A, B) The first two genotype MDS components for blood (A) and brain (B). (C, D) The first two gene expression PCA components on TMM normalized, log_2 transformed, OLS covariates corrected (sex, four genotype MDS, and dataset indicator variables), and log_2 mean and standard deviation returned data for blood (C) and brain (D). (E, F) The first two gene expression PCA components on TMM normalized, log_2 transformed, gene centered, sample z-score transformed, OLS covariates corrected (sex, four genotype MDS, and dataset indicator variables) data for blood (E) and brain (F).
