# Supplementary material for: PICALO: principal interaction component analysis for the identification of discrete technical, cell-type, and environmental factors that mediate eQTLs
Source: Genome Biol. 2024 Jan 22;25:29. doi: 10.1186/s13059-023-03151-0 (PMC10802033; doi:10.1186/s13059-023-03151-0)
Supplement: Supplementary file 5 — Additional file 5. Supplementary note. [file 13059_2023_3151_MOESM5_ESM.pdf]

Supplementary information

PICALO: principal interaction component analysis for the identification of discrete technical, cell-type, and environmental factors that mediate eQTLs

Martijn Vochteloo[1,2], Patrick Deelen[1,2], Britt Vink[1,3], BIOS Consortium, Ellen A. Tsai[4], Heiko Runz[4], Sergio Andreu-Sánchez[1,5], Jingyuan Fu[1,5], Alexandra Zhernakova[1], Harm-Jan Westra[1,2,6,✉], Lude Franke[1,2,6,✉]

1. Department of Genetics, University Medical Center Groningen, University of Groningen, Groningen, The Netherlands
2. Oncode Institute, Utrecht, The Netherlands
3. Institute for Life Science & Technology, Hanze University of Applied Sciences, Groningen, The Netherlands
4. Translational Sciences, Research and Development, Biogen, Cambridge, MA, USA
5. Department of Pediatrics, University Medical Center Groningen, University of Groningen, Groningen, Netherlands
6. These authors jointly supervised the work

✉ Corresponding authors: Harm-Jan Westra (h.j.westra@umcg.nl) and Lude Franke (l.h.franke@umcg.nl)

Table of Contents

PICALO: principal interaction component analysis for the identification of discrete technical, cell-type, and environmental factors that mediate eQTLs..................................1

Comprehensive simulation study shows that PICALO robustly identifies hidden eQTL contexts 2

Computational efficiency of PICALO 4

PICs identified with PICALO are robust with respect to the starting position used for optimization..5

Low ieQTL PICs are biologically relevant but should be interpreted with caution 7

BIOS Consortium (Biobank-based Integrative Omics Study) – Author information 8

Comprehensive simulation study shows that PICALO robustly identifies hidden eQTL contexts

In order to evaluate the performance of PICALO we employed a simulation study. While simulating eQTL effects are straightforward and widely applied, simulating interaction effects is much more challenging. Where biologically reliable eQTL betas in an eQTL simulation can simply be drawn from a normal distribution, interaction betas cannot. In practice the betas in an interaction model are dependent on each other and sampling each from a normal distribution could result in simulated expression for which the interaction term explains more variance than the main genotype effect. To circumvent this, we used real interactions observed with real genotype, expression, and covariate data as a template for generating biologically reliable interaction models. For simulating K hidden covariates, a template model for each ieQTL is constructed based on

$$M = 1 + genotype + X + X * genotype + noise \quad (1)$$

Where

- M is the interaction eQTL model matrix
- $\vec{1}$: a vector of ones of size n
- $genotype$: a vector of size n containing the real genotype data for this eQTL
- X: covariate matrix of size n by K where each covariate is normally distributed
- $X * genotype$: interaction effect between each covariate in X and the $genotype$

From this model we store the Minor Allele Frequency (MAF), beta's (β), standard deviations (σ) of all terms including the residuals. We then simulate a new genotype vector ($\widehat{genotype}$) with the observed MAF, and sample K covariates where each entry is drawn from $N(0, 1)$. Let m denote the

number of terms in the model and be equal to $3 + (2 * K)$. The ieQTL model (M) for the simulation is constructed as:

2 $$M = 1 + \widehat{genotype} + X_{simulated} + X_{simulated} * \widehat{genotype} + 1$$

The simulated expression is subsequently calculated by:

3 $$E = (R * \sigma + \beta) * M$$

Where

- R is a random matrix of size n by m, each entry is drawn from $N(0, 1)$.
- σ is the standard deviation vector of size m from the template model.
- β is the beta vector of size m from the template model.
- M is the simulated model matrix:

The resulting expression matrix E is summed per sample to create the simulated expression for gene g. A visual representation of this method is shown in Additional file 1: Fig. S19 and S20.

Computational efficiency of PICALO

PICALO is implemented efficiently, using limited memory and computing power requirements. We note that the total computation time highly depends on the number of eQTLs, samples, PICs, starting positions, and minimum number of optimization rounds. We simulated three hidden contexts using our blood dataset consisting of 13,059 eQTLs and 2,932 samples. We ran PICALO using 2 cores and observed that PICALO required on average 8 EM iterations to convergence, taking ±11 minutes per PIC. The maximum memory usage was on average 4.5Gb. The non-simulated results presented in this paper were generated enforcing a minimum of 50 optimization rounds which increased the computational burden considerably. Generating these results took 18 hours using 2 CPU cores and 5.3GB of RAM for blood (36 min/PIC), and 9 hours using 2 CPU cores and 3.8GB of RAM for brain (26 min/PIC).

PICs identified with PICALO are robust with respect to the starting position used for optimization

Since EM algorithms can yield results that depend on the expectation, in this case the choice of the starting position, we evaluated if the PICs were dependent on the starting position used for optimization (by default the starting position with most significant ieQTLs). For each of the first five PICs in blood, we reperformed the analysis while using each of the first 25 expression PCs as the starting positions for optimization and compared the PICs (henceforth referred to as outcomes). For PIC1 we found that irrespective of the starting position, all 25 PICALO analyses resulted in near-identical outcomes (average pair-wise Pearson r=0.9997; Additional file 1: Fig. S7A). We note that this was independent of the number of significant ieQTLs that was used to start the optimization (between 4 and 2,670, depending on the PC; Additional file 1: Fig. S7B). We then adjusted the expression data for PIC1 and its interaction effect with eQTLs and evaluated PIC2. Similarly, we observed a robust outcome of PICALO for 21 out of the 25 expression PCs used as starting position, with an average pair-wise Pearson r=0.9946. The remaining four PCs resulted in two distinct groups of PICALO outcomes: the first group (PC13 and PC20) had a Pearson r of 0.65 and <0.05 with the other outcomes, while the second group (PC14 and PC21) had a Pearson r of 0.44 and <0.01 with others. Each of these groups had a smaller number of significant ieQTLs than the original PIC2 (number of ieQTLs; PIC2 equivalents: 1,053 ±27, first group: 209 ±10, second group: 22 ±8). This observation confirms that, like other EM methods, PICALO may not always return the global optimum but may instead converge to a local optimum. Like for PIC1, we found no relationship between the number of eQTLs interacting with the starting position and the resulting PIC (Additional file 1: Fig. S7B). Since this step yielded multiple solutions, we selected the PIC with the largest number of ieQTLs before optimization as PIC2, identical to a normal PICALO procedure. For the subsequent PICs we observed that an increasing number of uncorrelated PICALO outcomes were identified dependent on the initial guess that was used for optimization. Notably, we observed that

the four local optimums identified in the PIC2 analyses are rediscovered as local optimums in PIC3, an example of which is shown in Additional file 1: Fig. S7C. In other words: the influence of the starting position on the resulting PIC increases as the local optimums in the data capture decreasing proportions of interaction variance. These observations suggest that PICs capture unique effects that can be robustly identified using PICALO, but that the order in which PICs are identified can be dependent on the starting position.

Low ieQTL PICs are biologically relevant but should be interpreted with caution

In this manuscript we restricted ourselves to PICs with at least 40 ieQTLs as our robustness analyses have indicated this to be the lower bound for replication. However, it can be that true eQTL context only affects a small set of genes, for example in a specific stimulation condition. Here we describe a few examples such PICs in blood and brain; yielding less than 40 ieQTL but having a notable biological signal. The annotation of these PICs should be interepreted with caution.

In blood we observed two PICs with a small number of eQTLs that showed high specificity for specific cell types. The negatively correlating genes for PIC26 (6 ieQTLs) and PIC29 (11 ieQTLs) both showed strong enrichment for B-cells (ToppCell enrichment p-value $<8.4\times10^{-173}$ for PIC26 and p-value $<6.2\times10^{-147}$ for PIC29). Moreover, single-cell expression showed that these genes are specifically expressed in B-cells, further confirming that these PICs capture B-cell differences.

In brain we also observed PICs that interact with only a limited set of eQTLs capturing less frequent cell types. For PIC13, interacting with 13 eQTLs, while we observed a low but significant correlation with predicted microglia proportion (Pearson r=0.08), we did observe a clear enrichment for microglia in the single-cell data, as well as an enrichment of microglial genes (ToppCell enrichment p-value $<1.4\times10^{-165}$). Finally, genes negatively correlating with PIC14, which interacts with 17 eQTLs, showed enrichment for endothelial cells in single-cell data as well as when using gene set enrichment (ToppCell enrichment p-value $<1.2\times10^{-51}$**)**. However, similar as PIC13, only a weak correlation was observed between PIC14 and the predicted endothelial cell counts (Pearson r=-0.07).

BIOS Consortium (Biobank-based Integrative Omics Study) – Author information

Management Team
Bastiaan T. Heijmans (chair)[1], Peter A.C. 't Hoen[2], Joyce van Meurs[3], Rick Jansen[5], Lude Franke[6].

Cohort collection
Dorret I. Boomsma[7], René Pool[7], Jenny van Dongen[7], Jouke J. Hottenga[7] (Netherlands Twin Register); Marleen MJ van Greevenbroek[8], Coen D.A. Stehouwer[8], Carla J.H. van der Kallen[8], Casper G. Schalkwijk[8] (Cohort study on Diabetes and Atherosclerosis Maastricht); Cisca Wijmenga[6], Lude Franke[6], Sasha Zhernakova[6], Ettje F. Tigchelaar[6] (LifeLines Deep); P. Eline Slagboom[1], Marian Beekman[1], Joris Deelen[1], Diana van Heemst[9] (Leiden Longevity Study); Jan H. Veldink[10], Leonard H. van den Berg[10] (Prospective ALS Study Netherlands); Cornelia M. van Duijn[4], Bert A. Hofman[11], Aaron Isaacs[4], André G. Uitterlinden[3] (Rotterdam Study).

Data Generation
Joyce van Meurs (Chair)[3], P. Mila Jhamai[3], Michael Verbiest[3], H. Eka D. Suchiman[1], Marijn Verkerk[3], Ruud van der Breggen[1], Jeroen van Rooij[3], Nico Lakenberg[1].

Data management and computational infrastructure
Hailiang Mei (Chair)[12], Maarten van Iterson[1], Michiel van Galen[2], Jan Bot[13], Dasha V. Zhernakova[6], Rick Jansen[5], Peter van 't Hof[12], Patrick Deelen[6], Irene Nooren[13], Peter A.C. 't Hoen[2], Bastiaan T. Heijmans[1], Matthijs Moed[1].

Data Analysis Group
Lude Franke (Co-Chair)[6], Martijn Vermaat[2], Dasha V. Zhernakova[6], René Luijk[1], Marc Jan Bonder[6], Maarten van Iterson[1], Patrick Deelen[6], Freerk van Dijk[14], Michiel van Galen[2], Wibowo Arindrarto[12], Szymon M. Kielbasa[15], Morris A. Swertz[14], Erik. W van Zwet[15,] Rick Jansen[5], Peter-Bram 't Hoen (Co-Chair)[2], Bastiaan T. Heijmans (Co-Chair)[1].

1. Molecular Epidemiology, Department of Biomedical Data Sciences, Leiden University Medical Center, Leiden, The Netherlands
2. Department of Human Genetics, Leiden University Medical Center, Leiden, The Netherlands
3. Department of Internal Medicine, ErasmusMC, Rotterdam, The Netherlands
4. Department of Genetic Epidemiology, ErasmusMC, Rotterdam, The Netherlands
5. Department of Psychiatry, VU University Medical Center, Neuroscience Campus Amsterdam, Amsterdam, The Netherlands
6. Department of Genetics, University of Groningen, University Medical Centre Groningen, Groningen, The Netherlands
7. Department of Biological Psychology, VU University Amsterdam, Neuroscience Campus Amsterdam, Amsterdam, The Netherlands
8. Department of Internal Medicine and School for Cardiovascular Diseases (CARIM), Maastricht University Medical Center, Maastricht, The Netherlands
9. Department of Gerontology and Geriatrics, Leiden University Medical Center, Leiden, The Netherlands
10. Department of Neurology, Brain Center Rudolf Magnus, University Medical Center Utrecht, Utrecht, The Netherlands
11. Department of Epidemiology, ErasmusMC, Rotterdam, The Netherlands
12. Sequence Analysis Support Core, Department of Biomedical Data Sciences, Leiden University Medical Center, Leiden, The Netherlands
13. SURFsara, Amsterdam, the Netherlands
14. Genomics Coordination Center, University Medical Center Groningen, University of Groningen, Groningen, the Netherlands
15. Medical Statistics, Department of Biomedical Data Sciences, Leiden University Medical Center, Leiden, The Netherlands
