# Supplementary material for: PICALO: principal interaction component analysis for the identification of discrete technical, cell-type, and environmental factors that mediate eQTLs
Source: Genome Biol. 2024 Jan 22;25:29. doi: 10.1186/s13059-023-03151-0 (PMC10802033; doi:10.1186/s13059-023-03151-0)
Supplement: Supplementary file 11 — Additional file 11. Review history. [file 13059_2023_3151_MOESM11_ESM.docx]

**1^st^ round**

**Reviewer 1**

The manuscript titled "Principal Interaction Component Analysis: Identification of Discrete Technical, Cell-Type, and Environmental Factors that Mediate eQTLs" addresses a concern in the analysis of context-specific effects of eQTL (or any other molecular QTL). The authors claim that existing hidden variable inference approaches for unobserved contexts are insufficient, and propose a new method called PICALO (Principal Interaction Component Analysis through Likelihood Optimization) to tackle the problem. To demonstrate the effectiveness of their approach, the authors applied PICALO to large-scale eQTL studies, namely BIOS (blood) and MetaBrain (brain). Their analysis led to the discovery of context-dependent eQTLs, and provided insights into the properties of the inferred hidden variables, including the interaction eQTLs (ieQTLs) as well as the additional ieQTLs identified with the new approach.


Overall, I find the problem itself is well posed and clearly described. The manuscript is also reasonably well written with a clear description of the PICALO approach using both text and figures. The application on such large scale bulk RNA-seq based eQTL study with knowledge from recent single cell work in brains is also impressive.

However, although I acknowledge and appreciate that unobserved variables (particularly biological variables) can hinder the discovery of context dependent QTL (dynamic QTL) which is of great interest in the molecular QTL research field, I am not entirely convinced that the PICALO idea is valid. In fact, I had the same doubt with the PEER factor analysis for hidden variable inference without considering interaction, that it is not justifiable to claim that PEER outperforms other approaches because it detects more eQTL. In a recent paper on Genome Biology which is also cited by this manuscript, Zhou and colleagues showed, and I quote, that "choosing the number of PEER factors by maximizing the number of discoveries (a common approach used by practitioners) may yield inappropriate choices of K, leading to model overfit and potential loss of power and precision". By the same token, maximizing the number of ieQTL discoveries can also lead to model overfit and potential loss of precision with many false positive results. PICALO is based on the exact scheme that identifies hidden variables by maximizing ieQTL. This can lead to systematic bias. I can imagine a scenario where the true ieQTL are strong and are localized to a small set of genes. Maximizing the discovery across the genome globally per sample as PICALO proposes would distort the true architecture of ieQTL.

Unlike Zhou et al, the authors did not perform any simulation to validate PICALO. Their validations rely on replication across studies and enrichment of ieQTL in functionally relevant contexts. But without comparisons to conventional approach for ieQTL with hidden covariates discovered by straightforward PCA or PEER analysis, I would argue that the signals might be more robust in the conventional approach compared to the new method. I believe the authors need substantial additional work to justify the validity of PICALO, and defining proper metric for performance comparison is a good starting point.


Minor comments:

Hardy-Weinberg exact test p-value cutoff is not consistent: both 0.001 and 1E-4 were proposed to use for QC. I suggest instead using a cutoff from bonferroni correction, ie 0.05 / number of variants analyzed.
Please comment on the computational efficiency and convergence properties of the proposed algorithm. The latter might be hard to achieve without a well designed simulation study.
Line 665 "In the AFR subset, we then used a dot product to associate the AFR gene expression levels with PIC-gene correlations observed in the EUR subset. This provides an estimate of the EUR PIC values in the AFR subset". EUR PIC values may have to do with technical variables specific to the EUR study and not applicable to the AFR subset. I am not sure what this is trying to capture.
Figure 1, does the numbers such as R2 mean anything specific, or just a generic illustration? If it is the latter I suggest removing those numbers to avoid confusions.
Line 158 " for instance for TUBB2A and C9orf78 (-log 10 p-value decrease 71.9 and 45.3)" -log10 p-value decrease means less significant discovery. Sounds like ieQTL based on PIC is less significant than based on PC?
Line 221 it would be good to show in comparison to conventional PC the correlation between technical covariates, in addition to that of PIC. Perhaps a conventional PC captures these known covariates better?
I struggle with what it means when a technical variable interacts with a SNP. For example, a SNP can have a smaller biological effect on gene expression in some samples with poor RNA quality, whereas the effect is larger for those with good RNA quality? More intuition on the implication of ieQTL with technical variables would be great.
322 "in de Klein" should remove the "de"?

Reference

1. Zhou et al, <https://genomebiology.biomedcentral.com/articles/10.1186/s13059-022-02761-4>

**Referee 2**

The manuscript by Vochteloo and colleagues presents PICALO, a method for unbiased discovery of latent contexts/environments and interaction eQTLs using an EM algorithm. PICALO is an unsupervised method to identify latent components, referred to as Principal Interaction Components (PICs), that interact with genetic effects on gene expression. The approach is intuitive: it mainly optimizes principal components to maximize the identification of interaction eQTLs (ieQTLs) via multiple iterations. The manuscript demonstrates the effectiveness of the novel approach using two large eQTL cohorts, blood (n=2,932) and brain(n=2,440). The manuscript shows how this new method can contribute to the discovery of distinct contexts through identification of the PICs, and has improved power compared to other existing approaches.
Despite these positive aspects, there are also some limitations, which are detailed in the following comments:

While the manuscript demonstrates that some of the identified PICs represent technical covariates such as RNAseq metrics, it would be useful to know the correlations of all biological variables and cell proportions to the identified PICs to understand how the PICs can capture different biological contexts.

The manuscript notes that this method could be applied to other datasets, however it is unclear whether this approach is applicable to datasets of different size, such as single cell RNA-seq data. More generally, it is important to include a broader discussion of limitations of the method.

The most interpretable PICs are those that capture cell proportions. It would be important to show how the results compare to the alternative approach of performing interaction-eQTL mapping when considering cell proportion inferred with other existing methods.

Biological interpretation of the PICs is not as clear cut as reported, so more caution in interpreting the PICs is needed.

**Authors’ response**

Summary of changes:

We would like to thank both reviewers for their valuable comments and suggestions. We believe we have fully answered the questions that have been raised and believe the comments have helped to considerably improve the manuscript. We have summarized the major changes below:

- We have updated our manuscript to conform to the style guide for Methods articles.
- We have now benchmarked our method using a synthetic dataset as suggested by reviewer 1.
- We have underlined the robustness of our results by performing a replication analysis of the PIC ieQTLs within each dataset by performing PIC discovery using eQTLs that map to odd chromosomes, and subsequently ascertaining the extent to which ieQTLs are identified using that PIC for genes that map to even chromosomes.
- We have expanded our systematic comparison between our method and conventional principal component analysis, as requested by reviewer 1.
- We have added additional supplementary tables that describe our comparison between PICs and technical or biological features, as requested by reviewer 2.
- We have performed a replication analysis of the cell context ieQTL effects by comparing them to bulk cell type proportion ieQTLs, as requested by reviewer 2.
- Finally, we have expanded the discussion section of our paper, clarifying any concerns the reviewers had.

One major aspect that both reviewers bring forward is a concern about the robustness of PICALO and its results. Through the changes above, this version of the manuscript now contains six different approaches that each highlight the robustness of PICALO: (1) replication with single-nucleus eQTLs from Bryois *et al.*, (2) replication in samples from a different ancestry, (3) within-dataset cross-validation through even / odd chromosome replication (reviewer 1 question 2), a simulation study (reviewer 1 question 4), (3) subsampling of datasets (reviewer 2 question 2), (4) and finally, replication with cell type ieQTLs from de Klein *et al.* (reviewer 2 question 3). Each of these approaches have shown good replication of our top PICs. We hope to have sufficiently convinced the reviewer of the robustness of our study.

We have provided answers to the individual questions of the reviewers below. Questions from reviewers are displayed in **bold**, responses in plain text, and changes to the manuscript in *italics*.

Response to referees

Reviewer #1

**Reviewer #1: The manuscript titled "Principal Interaction Component Analysis: Identification of Discrete Technical, Cell-Type, and Environmental Factors that Mediate eQTLs" addresses a concern in the analysis of context-specific effects of eQTL (or any other molecular QTL). The authors claim that existing hidden variable inference approaches for unobserved contexts are insufficient, and propose a new method called PICALO (Principal Interaction Component Analysis through Likelihood Optimization) to tackle the problem. To demonstrate the effectiveness of their approach, the authors applied PICALO to large-scale eQTL studies, namely BIOS (blood) and MetaBrain (brain). Their analysis led to the discovery of context-dependent eQTLs, and provided insights into the properties of the inferred hidden variables, including the interaction eQTLs (ieQTLs) as well as the additional ieQTLs identified with the new approach.**

**Overall, I find the problem itself is well posed and clearly described. The manuscript is also reasonably well written with a clear description of the PICALO approach using both text and figures. The application on such large scale bulk RNA-seq based eQTL study with knowledge from recent single cell work in brains is also impressive.**

We thank the reviewer for reviewing our manuscript. We have now addressed all concerns of the reviewers and improved the clarity and comprehensiveness of our work. We believe that our work presents an important approach to uncover the context-specificity of eQTL effects in bulk. As stated by the reviewer, better understanding the context dependency of eQTL effects is of great interest for the field and we appreciate that the reviewer underlines the relevance of our work.

**1. However, although I acknowledge and appreciate that unobserved variables (particularly biological variables) can hinder the discovery of context dependent QTL (dynamic QTL) which is of great interest in the molecular QTL research field, I am not entirely convinced that the PICALO idea is valid. In fact, I had the same doubt with the PEER factor analysis for hidden variable inference without considering interaction, that it is not justifiable to claim that PEER outperforms other approaches because it detects more eQTL. In a recent paper on Genome Biology which is also cited by this manuscript, Zhou and colleagues showed, and I quote, that "choosing the number of PEER factors by maximizing the number of discoveries (a common approach used by practitioners) may yield inappropriate choices of K, leading to model overfit and potential loss of power and precision". By the same token, maximizing the number of ieQTL discoveries can also lead to model overfit and potential loss of precision with many false positive results. PICALO is based on the exact scheme that identifies hidden variables by maximizing ieQTL. This can lead to systematic bias. I can imagine a scenario where the true ieQTL are strong and are localized to a small set of genes. Maximizing the discovery across the genome globally per sample as PICALO proposes would distort the true architecture of ieQTL.**

We fully agree with the reviewer that maximizing the number of interaction eQTL (ieQTL) discoveries could indeed lead to model overfit. However, the number of significant ieQTLs is a determining factor only at the first step of PICALO, when the program decides which principal component to use as a starting vector for the PICALO algorithm. First, PICALO maximizes the joint likelihood of the interaction effect for the set of eQTLs that show a significant interaction effect with this component, by adjusting the values of the chosen principal component. Then, PICALO determines whether this updated context results in more significant interaction effects for other eQTLs as well. As long as there is a change observed in the optimized context, the procedure is repeated. However, it is also a possibility that the number of significant ieQTLs decreases after optimization. As such PICALO does not maximize the number of ieQTL discoveries, but it maximizes the interaction variance for a set of ieQTLs.

We do acknowledge though that this leads to PICs that can show some overfitting, since PICALO adjusts the vector in such a way that the interaction variance is maximized for the set of ieQTLs under investigation. We have now systematically studied the potential for overfitting, by first running PCA and PICALO on only expression data from genes (and thus *cis-*eQTLs) that map to odd chromosomes. Once PICALO had completed (while only using eQTLs on odd chromosomes), we studied to what extent the PICs, identified using data from only the odd chromosomes, showed interaction effects with eQTLs that map to even chromosomes. This permitted us to ascertain to what extent the PICs reflect genuine technical or biological factors that also show interaction effects on eQTLs that map to even chromosomes or whether these PICs reflect overfitted effects, specific to the included eQTLs on the odd chromosomes (rebuttal figure 1). We describe these results in detail in our response to the second question of reviewer 1. In short, our new results indicate that, when PICALO has completed, the PICs that show interaction effects with at least 40 eQTLs that map to odd chromosomes, also show consistent interaction effects on eQTLs that map to even chromosomes.

When comparing expression PC1 and PIC1 (which both captures sample quality), we also observed that PIC1 yields a substantially higher number of significant ieQTLs, even when PIC1 is identified using eQTLs on odd chromosomes and the number of significant interaction effects is determined for eQTLs that map to even chromosomes. In blood, using only information from odd chromosomes, expression PC1 yielded 1,184 significant ieQTLs that map to even chromosomes whereas PIC1 (detected using only information from odd chromosomes) yielded 1,515 significant ieQTLs that map to even chromosomes reflecting a 28% increase. For brain, these numbers are 1,140 and 441, reflecting a 258% increase (rebuttal figure 1).

Expression PC1, detected on non-centered gene expression data (in contrast to the more common strategy of conducting PCA on centered gene expression data) captures sample quality. As expected, we subsequently observe many significant ieQTLs for expression PC1 with many eQTLs. However, when using expression PC1 as a start for its algorithm, we do observe that PICALO identifies a PIC that results in many additional ieQTLs. We reason this is unlikely to be attributed to overfitting since we used odd chromosomes for PIC discovery and even chromosomes for benchmarking the number of identified ieQTLs. This suggests that there may be other factors apart from RNA quality that influence effect sizes of many eQTLs. To our knowledge it has not been previously described in literature that RNA quality shows an interaction effect with thousands of eQTLs. We believe that researchers who are interested in performing ieQTL analysis in bulk datasets should be aware of this observation, and should first correct eQTLs for interaction effects with RNA-quality, either based using PCA(determined on non-centered gene expression data), or, ideally, by using PICALO. This correction might in turn also improve downstream comparisons with GWAS signals such as colocalization and Mendelian Randomization.

Finally, we agree with the reviewer that biologically driven ieQTL (e.g. cell-type dependent ieQTLs) are limited to a smaller set of genes. We have now added this to the discussion as follows:

*“Overall, the purpose of PICALO is to identify hidden contexts that mediate eQTL effect sizes. As reported before, we have shown that cell type proportion is an important biological context to consider. However, although eQTLs are plentiful, our results suggest that only a modest proportion of eQTLs identified in bulk data are context specific for biological contexts. This could be because cell type regulatory effects can be shared between cell types from the same lineage, or because they could be dependent on extracellular stimulation. We therefore underline that even after PICALO optimization we expect the number of true interaction QTLs to be limited to a small set of genes.*“

Considering that many of the detected ieQTLs may be due to RNA-quality differences, our findings underline that even context localized to a small set of genes can be detected and can be highly relevant. However, we acknowledge that this does not apply for all PICs we have identified. It remains to be seen whether our inability to satisfactorily annotate some of our PICs is due to a lack of knowledge of cell specific signals, lack of sample metadata (e.g.: technical and environmental factors, health information, biopsy details), or whether we are picking up on technical noise. As such, we highlighted this limitation in our discussion as follows:

*“PICALO has a number of limitations. Like any hidden variable inference method, the identified PICs do not have a direct functional annotation and therefore require further analyses for proper interpretation. While we have shown that his is feasible, we acknowledge that the confidence in the annotation is dependent on the quality and availability of ground truth information such as gene pathways, cell type specificity of genes, as well as phenotypic and technical covariates in the dataset. As a result, the interpretation of PICs interacting with only a small set of eQTLs should be done with caution.”*

**2. Unlike Zhou et al, the authors did not perform any simulation to validate PICALO. Their validations rely on replication across studies and enrichment of ieQTL in functionally relevant contexts. But without comparisons to conventional approach for ieQTL with hidden covariates discovered by straightforward PCA or PEER analysis, I would argue that the signals might be more robust in the conventional approach compared to the new method. I believe the authors need substantial additional work to justify the validity of PICALO, and defining proper metric for performance comparison is a good starting point.**

To demonstrate that PICALO provides substantial additional knowledge of eQTL context not captured by PCA, we have systematically compared our PICs to straightforward PCA components in the paragraph ‘PICs capture more interaction variance using fewer components than PCA’. We chose not to run PEER analysis, since Zhou *et al.* showed that PCA outperforms PEER. In figure 2G, we demonstrate that PICs are better able to distinguish between technical and biological context than PCA, where only a limited number of PICs correlate with known technical confounders while all PCA components appear to be affected (we made a similar observation in brain; supplementary figure 6B). As a result, many of the ieQTLs reported when using principal components as context could be influenced by technical confounding. In contrast, PICALO only requires a handful of PICs to capture all technical interaction variance allowing for a more biological relevant signal with clear annotation in the remaining PICs.

We now also studied the robustness of PICALO, by splitting the two expression datasets in a discovery and replication dataset: we generated an expression dataset that comprises only genes that map to odd chromosomes, used for discovering gene expression PCs and PICs and an expression dataset of genes that map to even chromosomes, used for ascertaining the number of ieQTLs that can be identified using the PCs and PICs that were identified on the odd chromosomes (rebuttal figure 1; Fig. S8). This analysis is more extensively introduced in reviewer 1 question 1. We observed that the total number of interactions was comparable between expression PCs and PICs. However, PICs captures more interactions within the first components, interact with more unique eQTLs (2% additional in blood and 6% additional in brain), and shows higher specificity for eQTLs only interacting with one PIC (8% additional in blood and 17% additional in brain). While both PICs and expression PCs show good replication, the increased specificity of PICs adds additional value in the interpretation of eQTLs contexts as highlighted throughout the manuscript.

We have summarized these results in the manuscript as follows:

*“We then further assessed the robustness of the identified PICs in blood and brain by splitting each expression matrix in genes that map to odd chromosomes (discovery) and genes that map to even chromosomes (replication). We then identified expression PCs and PICs using the odd chromosome (eQTL) genes and subsequently determined how many ieQTLs could be identified with those components on the even chromosome eQTLs. Generally, we observed that PICs with at least 40 ieQTLs replicated in the odd chromosome eQTLs. In blood we identified 6 PICs with at least 40 ieQTLs in the odd chromosome eQTLs with which 1,658 unique even chromosome eQTLs showed a significant interaction (31%). Comparatively, the same number of expression PCs interacted with 1,552 unique even chromosome eQTLs. In brain, the difference was even larger with 1,376 unique even chromosome eQTLs showing a significant interaction compared to 1,105 for the same number of expression PCs. Most notably, PICs show a higher specificity than expression PCs by only interacting with one gene in 82% of cases compared to 74% for expression PCs in blood and 88% compared to 61% in brain (Fig. S8).”*

Rebuttal figure 1. Flowchart indicating discovery and replication scheme to test the robustness of PICALO results. This figure is also added to the manuscript as Fig. S8.

However, we acknowledge that the rate of replication of PICs is low when the number of ieQTLs is small and we therefore advise caution when interpreting these findings. Therefore, we have moved all biological interpretation of PICs with less than 40 ieQTLs to the Supplementary Note:

*“For the interpretation of the non-technical PICs we confined ourselves to those PICs with a minimum of 40 ieQTLs based on our simulation analyses (first 10 PICs in blood, first 7 in brain). We note however that true eQTL context may interact with only a handful of genes and therefore highly informative biological context might be overlooked (Supplementary Note).”*

We have also added a warning in the discussion:

*“As a result, the interpretation of PICs, interacting with only a small number of eQTLs, should be done with caution.”*

**Minor comments:**

**3. Hardy-Weinberg exact test p-value cutoff is not consistent: both 0.001 and 1E-4 were proposed to use for QC. I suggest instead using a cutoff from bonferroni correction, ie 0.05 / number of variants analyzed.**

We thank the reviewer for pointing out this oversight in consistency in our thresholding. Generally, variants are selected to be included in a genetic analysis when they have a Hardy-Weinberg p-value (HWE-P) above a certain HWE P-Value threshold. Consequently, lower HWE-P-Value thresholds would result in the inclusion of additional variants. The metric indicates whether the observed genotype distribution is similar to the expected genotype distribution given the sample size and allele frequency. Deviations from Hardy-Weinberg equilibrium can occur (amongst others) because of technical artifacts during genotype calling, particularly when the allele frequencies are low, when SNPs map in a structural variant, or when the genotype data consists of individuals from different ancestries. As such, when calculating genotype MDS components, an argument could be made that adding variants with lower HWE-P values would be beneficial, because it could make the resulting MDS components better able to capture population stratification or genotyping differences, while at the same it could deteriorate such MDS when SNPs are included with incorrectly called genotypes.

To calculate genotype MDS components we excluded variants with an HWE-P lower than 1x10^-3^. This threshold is substantially stricter than the Bonferroni cutoff proposed by the reviewer (0.05/8,138,962 = 6.1x10^-9^ for blood and 0.05/9,038,924 = 5.5x10^-9^ for brain). To test the consequences on the resulting MDS components, we applied the proposed Bonferroni threshold to the blood dataset, recalculated the MDS components, and correlated them with the previous MDS components. After pruning, the Bonferroni corrected MDS components we calculated based on 8,654 additional variants. We observed no notable difference in the resulting MDS components except for a few specific samples (visible in rebuttal figure 2 panel C and panel D). However, these samples are flagged as outliers in both analyses and removed in the subsequent QC step.

Rebuttal figure 2. Comparison of MDS components with 1x10^-3^ (y-axis) and Bonferroni threshold 6.1x10^-9^ (x-axis) in blood.

For the subsequent eQTL analyses, we applied a HWE p-value threshold of 1x10^-4^ which is slightly stricter than the Bonferroni cutoff proposed by the reviewer (0.05/13,058 = 3.8x10^-6^ for blood and 0.05/11,312 = 4.4x10^-6^ for brain). In eQTL or GWAS analysis, 1x10^-4^ is commonly used (e.g.: de Klein *et al.*, Nature Genetics 2023), and applying a Bonferroni correction would only result in a limited increase in the number of tested eQTLs (3 additional tested eQTLs in blood and 26 additional in brain). We therefore conclude that decreasing our HWE threshold has no influence on the results presented in the paper.

**4. Please comment on the computational efficiency and convergence properties of the proposed algorithm. The latter might be hard to achieve without a well designed simulation study.**

We thank the reviewer for this suggestion. We have now performed simulations to assess to what extent unobserved contexts can be identified by PICALO, when the initial correlation of the expression components with these unobserved contexts was limited. We determined the computational efficiency (i.e.: the memory / CPU usage and the number of iterations for the PICALO algorithm until convergence) and determined whether the resulting PICs showed a strong correlation to the simulated contexts.

We simulated eQTL interactions using three different contexts and modelled the simulated data according to what we had observed in the blood cohort: We simulated main eQTL effects for 13,059 *cis-*eQTLs, using a main genotype effect-size and minor allele frequency as we had observed per *cis*-eQTL in the blood data. For each eQTL, we also modelled a context and interaction effect with the context and main context effect, using the first three expression PCs forced into a normal distribution per dataset by ranking with ties (i.e.: mean = 0, standard deviation = 1) as observed in the blood data. To generate the three simulated contexts we randomly sampled from a normal distribution with mean 0 and standard deviation 1 (rebuttal figure 3A and B). 

As starting vector for PICALO optimization we simulated three random starting vectors that showed a certain correlation with the simulated contexts, ranging from 0 to 1.0 in increasing steps of 0.1. We then ran PICALO on each set of starting vectors, permitting us to ascertain for each of these starting vectors to what extent PICALO yielded PICs that reflected the simulated contexts.

For PIC1, affecting 3,214 ieQTLs (blue line in rebuttal figure 3C), we observe that the correlation of the starting vector with the actual simulated context can be very small: even when the initial correlation with this context is only 0.2 (rebuttal figure 3D, left), PICALO is able to correctly infer the simulated context (rebuttal figure 3D, right, r=0.96). PICALO needs five iterations of the EM algorithm to do so.

To a considerable extent this is also the case for PIC2 (the green line in rebuttal figure 3C), that affects approximately 920 ieQTLs: when the correlation of the starting vector with the simulated context is 0.4 (rebuttal figure 3E, left), PICALO is able to infer the actual simulated context (rebuttal figure 3E, right, r=0.76). PICALO needs 17 iterations of the EM algorithm to do so. For PICs that affect fewer ieQTLs (e.g.: PIC3), we observed that the better the correlation of the start vector with the simulated context, the more ieQTLs are identified and the better PICALO is able to reconstruct the hidden context. For PIC3 an initial correlation of 0.7 with the actual context is required to achieve a substantial number of ieQTLs to converge. These results thus indicate that PICALO is successful in identifying hidden contexts, provided that the number of ieQTLs that are affected by this context is substantial, or that the hidden context is already to a considerable extent captured by principal components.

Rebuttal figure 3. (A, B) Flowchart indicating discovery and replication scheme to simulate interaction eQTL data. (A) We generate a template model using real data from which the effect sizes, standard deviations, and minor allele frequency (MAF) are used to generate simulated data. (B) Genotypes are randomly generated using the observed minor allele frequency, 3 random covariates are sampled from a normal distribution and are consistent over all simulated eQTLs. The expression is then calculated by a weighted multiplication of the simulated model by the observed effect sizes and standard deviations. The dots indicate the example visualized in panel D (blue) and E (green). (D, E) Comparison between the start vector (left), identified PIC (right) and the simulated context (y-axis) for PIC1 using a starting vector of ±0.2 (D). (E) similar as (D), but for PIC2 using a starting vector of ±0.4. The r value denotes the Pearson correlation. This figure is also included in the manuscript as Fig. S5.

We have summarized this analysis in the manuscript results section as follows:

*“In order to evaluate to what extent PICALO is able to reconstruct hidden covariates we performed a simulation study. Using the effect sizes as observed in the real data, we simulated expression data containing a set of hidden interaction covariates. PICALO is able to robustly and efficiently identify simulated interaction context even if the starting vector has minimal correlation with the actual context (r ~0.2; Supplementary Note). However, PICALO’s reconstruction performance decreases if the starting vector does not interact with a sufficient amount of ieQTLs (Fig. S5; Supplementary Note).”*

We have provided detailed information of our methodology of generating simulated expression data in the Supplementary Note. Furthermore, we have recorded the computational burden of PICALO while running simulations and have summarized this information in the Supplementary Note as follows:

*“PICALO is implemented efficiently, using limited memory and computing power requirements. We note that the total computation time highly depends on the number of eQTLs, samples, PICs, starting positions, and minimum number of optimization rounds. We simulated three hidden contexts using our blood dataset consisting of 13,059 eQTLs and 2,932 samples. We ran PICALO using 2 cores and observed that PICALO required on average 8 EM iterations to convergence, taking ±11 minutes per PIC. The maximum memory usage was on average 4.5Gb. The non-simulated results presented in this paper were generated enforcing a minimum of 50 optimization rounds which increased the computational burden considerably. Generating these results took 18 hours using 2 CPU cores and 5.3GB of RAM for blood (36 min/PIC), and 9 hours using 2 CPU cores and 3.8GB of RAM for brain (26 min/PIC).”*

**5. Line 665 "In the AFR subset, we then used a dot product to associate the AFR gene expression levels with PIC-gene correlations observed in the EUR subset. This provides an estimate of the EUR PIC values in the AFR subset". EUR PIC values may have to do with technical variables specific to the EUR study and not applicable to the AFR subset. I am not sure what this is trying to capture.**

We apologize for having been unclear here. The samples in the AFR and EUR datasets were derived from the same brain cohorts. As such, we would expect that the RNA-seq measurements for these datasets could be affected by similar technical biases. The purpose of this analysis was therefore to show that the PICs produced by PICALO are not random, but rather give similar results when tested in a similarly processed dataset: eQTLs that show interactions with PICs in samples from EUR ancestry also show these interactions when we test these eQTLs in a replication cohort, consisting of samples from AFR ancestry. We have highlighted this as follows:

*“Given that the AFR samples originate from cohorts that also include EUR samples, we expect to replicate both biological as well as technical contexts in the AFR samples.”*

**6. Figure 1, does the numbers such as R2 mean anything specific, or just a generic illustration? If it is the latter I suggest removing those numbers to avoid confusions.**

We agree with the reviewer and have removed the R^2^ values for the cartoon illustrations. We have kept the summary statistics for the figure on the bottom right, illustrating a relationship between PIC2 and myeloid proportion, since this was generated using actual data.

**7. Line 158 “ for instance for TUBB2A and C9orf78 (-log 10 p-value decrease 71.9 and 45.3)” -log10 p-value decrease means less significant discovery. Sounds like ieQTL based on PIC is less significant than based on PC?**

We thank the reviewer for pointing out this error. The text now reads:

*“After applying PICALO we observed a large increase in the significance of the interaction term for a large proportion of ieQTLs, for instance for TUBB2A and C9orf78 (-log_10_ p-value increase 71.9 and 45.3) in blood (Fig. 2A) and FAM221A and ADAMTS18 (-log_10_ p-value increase 41.5 and 49.3) in brain (Fig. 2B).”*

**8. Line 221 it would be good to show in comparison to conventional PC the correlation between technical covariates, in addition to that of PIC. Perhaps a conventional PC captures these known covariates better?**

We thank the reviewer for this suggestion. In rebuttal figure 4 we show the correlations between PIC1 and expression PC1 for both blood and brain. Indeed, expression PC1 also captures the within-dataset correlation with our proxy for RNA-seq sample quality. However, this is to be expected since PIC1 is identified by optimization of the interactions as observed with expression PC1. As a result, we agree with the reviewer that conventional expression PCs are sufficient in identifying the first technical confounder. However, as mentioned in our response to reviewer 1 question 2, we do observe that PIC1 shows significant interaction effects for many more eQTLs, as compared to the number of significant interaction effects for expression PC1, both in blood and in brain. This indicates that while RNA quality has a substantial effect on eQTL effect sizes, and PC1 and PIC1 both capture this, PIC1 reveals many more eQTLs that have a significant interaction effect with RNA quality. We included this in the manuscript as follows:

*“We further observed that PIC1 shows significant interaction effects for many more eQTLs, as compared to the number of significant interaction effects for expression PC1, both in blood and in brain. This indicates that while RNA quality (captured by both PIC1 and PC1) has a substantial effect on eQTL effect sizes, PIC1 reveals many more eQTLs that have a significant interaction effect with RNA quality.”*

Rebuttal figure 4. Regression plot showing the correlation between PIC1 (A and B) and expression PC1 (C and D) and estimated RNA-seq sample quality calculated as the per sample expression correlation with the overall average expression in blood and brain.

**9. I struggle with what it means when a technical variable interacts with a SNP. For example, a SNP can have a smaller biological effect on gene expression in some samples with poor RNA quality, whereas the effect is larger for those with good RNA quality? More intuition on the implication of ieQTL with technical variables would be great.**

We fully agree with the reviewer that the effect on gene expression with samples with poor RNA quality is noisier as compared to samples with good RNA quality. This is exactly what we observe in the data, which shows that 88% of ieQTLs with PIC1 in both blood and brain have an increased effect size in high quality samples. We have now discussed these findings in our results:

*“We further observed that 88% of eQTLs that interact with PIC1 have a positive interaction effect, suggesting an increased effect size in high quality samples, both in blood and in brain.”*

Effect estimates determined by linear regression in real data can be biased by technical confounders. For instance, in the case of standard QTL analysis, PEER factors or PCs are often added to models to account for (hidden) confounders, which may consequently increase or decrease the observed effect size estimates (and standard errors) for the genotype effect. We reason this is no different for interaction effects estimated through linear regression: one way of thinking about this is that when a sample has a lower RNA quality, the estimation of the genotypic effect on measured gene expression levels may be less clear, as compared to a sample that has a higher RNA quality. The resulting variability in effect size estimates, dependent on RNA quality, could therefore result in an observed interaction. In other words: part of the observed interaction effect estimate may in fact not be due to a true biological effect, but rather because of bias of a (technical) confounder.

We have now described this intuitive explanation in the discussion section as follows:

*“In this manuscript we have highlighted a possible confounding effect of technical variation on the detected interaction eQTLs. Although RNA-seq quality metrics or expression PCs are usually considered as confounders, their interactions with genotype are, to our knowledge, never accounted for. However, such effects could be observed when samples with lower quality have increased error margin in their measured expression compared to samples with higher quality. For instance, when the samples would be stratified on RNA-quality, this could result in different observed eQTL effect sizes, because of differences in eQTL detection power between high quality and low-quality samples, in turn resulting in an interaction effect.”*

**10. 322 "in de Klein" should remove the "de"?**

“de Klein” is a common Dutch family name. Since ‘de’ is a Dutch article, according to Dutch spelling rules, it should not start with a capital ‘D’. We fully agree with the reviewer this is quite odd. This might be one of several reasons why we did not choose a profession in Dutch linguistics.

**Reference**

**1.** [**Zhou et al,**](https://eur03.safelinks.protection.outlook.com/?url=https%3A%2F%2Fgenomebiology.biomedcentral.com%2Farticles%2F10.1186%2Fs13059-022-02761-4&data=05%7C01%7Cm.vochteloo%40umcg.nl%7C8eed0aec1c7b4140b49d08db4bd638ef%7C335122f9d4f44d67a2fccd6dc20dde70%7C0%7C0%7C638187155691020784%7CUnknown%7CTWFpbGZsb3d8eyJWIjoiMC4wLjAwMDAiLCJQIjoiV2luMzIiLCJBTiI6Ik1haWwiLCJXVCI6Mn0%3D%7C3000%7C%7C%7C&sdata=aER2vDizKDdFCRqTmf3paPgv6GoPgUCUqw7MCrTqtc4%3D&reserved=0)

Reviewer #2

**Reviewer #2: The manuscript by Vochteloo and colleagues presents PICALO, a method for unbiased discovery of latent contexts/environments and interaction eQTLs using an EM algorithm. PICALO is an unsupervised method to identify latent components, referred to as Principal Interaction Components (PICs), that interact with genetic effects on gene expression. The approach is intuitive: it mainly optimizes principal components to maximize the identification of interaction eQTLs (ieQTLs) via multiple iterations. The manuscript demonstrates the effectiveness of the novel approach using two large eQTL cohorts, blood (n=2,932) and brain(n=2,440). The manuscript shows how this new method can contribute to the discovery of distinct contexts through identification of the PICs, and has improved power compared to other existing approaches.**

We thank the reviewer for reviewing our manuscript.

**Despite these positive aspects, there are also some limitations, which are detailed in the following comments:**

**1. While the manuscript demonstrates that some of the identified PICs represent technical covariates such as RNAseq metrics, it would be useful to know the correlations of all biological variables and cell proportions to the identified PICs to understand how the PICs can capture different biological contexts.**

We thank the reviewer for this suggestion. For our annotation we have, amongst other things, correlated the PICs will all phenotypes we had available for these samples. This includes dataset indicator variables, RNA-seq quality metrics, sex, genotype MDS components, expression PCs, measured / predicted cell proportions, measured blood statistics (HCT, HGB, MCH etc.), RNA ratio, LDL cholesterol, age, weight, smoking status, etc. We have now added these comparisons as supplementary tables 4 and 5 to the manuscript.

**2. The manuscript notes that this method could be applied to other datasets, however it is unclear whether this approach is applicable to datasets of different size, such as single cell RNA-seq data. More generally, it is important to include a broader discussion of limitations of the method.**

To evaluate the applicability of our method on datasets of different sizes we have evaluated the number of detected ieQTLs using a systematically increasing sample size subset of our blood dataset (rebuttal figure 6, Fig S6). We observed a near linear relationship between the sample size, the effect size of the context, and the number of significant ieQTLs. Given these effect sizes, these results indicate that a sufficient number of significant ieQTLs are found for PICALO to yield reliable PICs when the number of samples is approximately 1,000 or more.

Rebuttal figure 5. Comparison of the influence of dataset sample size on the number of detected ieQTLs using expression PCs as covariates in blood (A) and brain (B). This figure is also included in the manuscript as Fig. S6.

Moreover, as mentioned in our answer for reviewer 1 question 4, we have now conducted a simulation study to determine the convergence properties of our method. We observed that PICALO requires a minimum number of significant ieQTLs at the start, to reconstruct the originally simulated context. As a result, we argue that PICALO can be applied to smaller datasets, provided that contexts exist in the data that affect a substantial number of eQTLs. PICALO can also be applied to single-cell data when the number of donors is sufficiently large and pseudobulk data is used as input for PICALO. We believe that PICALO is particularly suited for upcoming, larger molecular QTL datasets. We expect that eQTL datasets will increase substantially in size in the near future, where PICALO will benefit from having more significant ieQTLs to start with. We expect this will result in improved power to infer more hidden contexts, with higher accuracy.

We have now discussed these findings in our results:

*“Moreover, we down sampled both the brain and blood eQTL datasets and observed that the power to detect ieQTLs is dependent on factors such as sample size, the number of eQTLs affected by specific contexts, and the overall effect sizes of these interactions (Fig. S6).”*

We have now also extended the discussion on the limitations of the method by adding the following points:

*“Moreover, the performance of PICALO is dependent on factors such as sample size, the number of eQTLs affected by specific contexts, and the overall effect sizes of these interactions. We envision that sample sizes of molecular QTL datasets will increase substantially in the near future. PICALO is particularly well suited to such larger datasets, because it will benefit from having more significant ieQTLs to start with, resulting in improved power to increase the number and accuracy of identified hidden contexts.”*

**3. The most interpretable PICs are those that capture cell proportions. It would be important to show how the results compare to the alternative approach of performing interaction-eQTL mapping when considering cell proportion inferred with other existing methods.**

We agree with the reviewer that PICs describing biological contexts are the most interpretable. While we acknowledge the relevance of comparing PIC ieQTLs with cell proportion ieQTLs, we wish to emphasize that PICALO’s purpose is to find shared biological context affecting eQTLs. As a result, PICs also capture shared effects within cell lineages in contrast to cell proportion ieQTLs. Therefore, a comparison of performance is not applicable. Regardless, we have replicated our brain PIC ieQTLs in the cell proportion eQTLs as reported by de Klein *et al.* 2023 using the same samples. We highlight results of three PICs in rebuttal figure 6 and provide a full version added as Fig. S17.

Rebuttal figure 6. *Replication of MetaBrain Cortex-EUR PIC ieQTLs in the MetaBrain cell type ieQTLs by Klein et al. Each column denotes a different PIC, each row compares the PIC ieQTLs to the eQTLs identified in a distinct cell type. Cell types are abbreviated as follows: AST = astrocytes, END = endothelial cells, EX = excitatory neurons, IN = inhibitory neurons, MIC = microglia, OPC = oligodendrocytes, NEU = other neurons. The shaded area denotes the 95% confidence interval. Each point denotes an eQTL of which the x-axis denotes the log interaction beta with a certain PIC, the y-axis denotes the log eQTL beta, and the color denotes if the eQTL effect significantly replicates (BH-FDR ≤ 0.05). Only significant interaction eQTLs are shown. The legend shows the sample size, Pearson correlation coefficient, the allelic concordance, the R_b_ and finally the π_1_ statistics. A full version of this figure is supplied with the manuscript as Fig. S17.*

The results very closely recapitulate the replication result in the Bryois *et al.* single-nucleus eQTLs in our manuscript. Note the strong shared effects over all cell types for PIC1, the higher correlation of effect sizes for glial cells and most notably oligodendrocytes for PIC2, and finally for PIC5 an astrocyte specific effect, all in line with our assigned labels. This was expected since de Klein *et al.* reported high replication between the cell proportion eQTLs and the same Bryois *et al.* single-nucleus eQTLs we used for replication. However, we note that these cell proportion eQTLs are enriched for cell type specific effects and fail to identify shared effects between cell types due to the method applied. As a result, we consider the Bryois *et al.* single-nucleus eQTLs as ground truth for our replication. We have included this analysis in the manuscript as another source of support for our PIC annotation in brain.

**4. Biological interpretation of the PICs is not as clear cut as reported, so more caution in interpreting the PICs is needed.**

We have updated the discussion as follows:

*“PICALO has a number of limitations. Like any hidden variable inference method, the identified PICs do not have a direct functional annotation and therefore require further analyses for proper interpretation. While we have shown that his is feasible, we acknowledge that the confidence in the annotation is dependent on the quality and availability of ground truth information such as gene pathways, cell type specificity of genes, as well as phenotypic and technical covariates in the dataset.”*

**2^nd^ round**

**Reviewer 2**

This revised version addresses my previous comments.
